# Random selection of factors preserves the correlation structure in a linear factor model to a high degree

**DOI:** 10.1371/journal.pone.0206551

**Published:** 2018-12-21

**Authors:** Antti J. Tanskanen, Jani Lukkarinen, Kari Vatanen

**Affiliations:** 1 Confederation of Finnish Industries EK, Helsinki, Finland; 2 Department of Mathematics and Statistics, University of Helsinki, Helsinki, Finland; 3 Varma Mutual Pension Insurance Company, Helsinki, Finland; Universita degli Studi del Piemonte Orientale Amedeo Avogadro, ITALY

## Abstract

In a very high-dimensional vector space, two randomly-chosen vectors are almost orthogonal with high probability. Starting from this observation, we develop a statistical factor model, the random factor model, in which factors are chosen stochastically based on the random projection method. Randomness of factors has the consequence that correlation and covariance matrices are well preserved in a linear factor representation. It also enables derivation of probabilistic bounds for the accuracy of the random factor representation of time-series, their cross-correlations and covariances. As an application, we analyze reproduction of time-series and their cross-correlation coefficients in the well-diversified Russell 3,000 equity index.

## 1 Introduction

### 1.1 Vectors in a high-dimensional space

In a high-dimensional vector space, any two unit-length random vectors with random independent components are typically nearly orthogonal with respect to each other [1, 2]. To have a concrete example, one could consider random vectors in $R^{d}$ whose components are taken independently from a common distribution with a zero mean and a variance 1/*d*. Then it is straightforward to check that the expectation of the square of the length of a vector is one and the expectation of a scalar product between any two independently chosen vectors is zero.

Mathematical probability theory has techniques which allow deriving explicit estimates for correlations of such high dimensional random vectors and exploring their dependence on the random distribution. Detailed examples will be given later and further discussion may be found in S1 Appendix. For example, it is possible to check whether the expected length squared and scalar products in the above random vector example are reached with a high probability. If the common distribution is Gaussian, we may conclude from the results given in Section 2.4.3 that the variances of both of observables are *O*(1/*d*) as *d* → ∞. By Chebyshev’s inequality, these estimates imply that the respective means are reached up to some fixed accuracy with a probability 1 − *O*(1/*d*) which thus occurs nearly always if *d* is sufficiently large. Please see https://github.com/ajtanskanen/Random-selection-of-factors-demo for a numerical demonstration.

The maximal number of nearly orthogonal unit vectors is closely related to generic geometric properties of high-dimensional spaces. For instance, even though the maximal number of exactly orthogonal unit vectors from $R^{d}$ is *d*, the maximal number of nearly orthogonal unit vectors grows exponentially fast with *d*. More precisely, suppose we call two unit vectors *ε*-quasiorthogonal if the absolute value of their inner product is at most *ε* for some *ε* > 0. Then the maximal size of a collection of *ε*-quasiorthogonal vectors in $R^{d}$ grows at least exponentially fast in *d* for any fixed *ε* > 0, as proven in [3].

These observations have relevance to time-series analysis, since a long time-series corresponds to a vector in a high-dimensional vector space and orthogonality of vectors corresponds to uncorrelatedness of time-series. Time-series corresponding to two randomly selected vectors are therefore expected to be in general almost uncorrelated. When the length of the time-series increases, or equivalently, dimension of the corresponding vector space increases, the probability that two randomly-selected time-series are uncorrelated increases [1]. If we select a set of, say, *n* vectors randomly, these vectors are approximately orthogonal to each other if the dimension of the space is sufficiently high. Here, high-dimensionality of the data may even be an asset: in a high-dimensional space, almost any set of random vectors yields an almost uncorrelated set of factor time-series that can be used as a basis for a linear factor model.

### 1.2 Factor models

Factor models are extensively used in financial applications to model asset returns [4] and to decompose them to loadings of risk factors. Factor models have provided insight into, e.g., the drivers of asset returns, and they have influenced the way risks are managed. In short, factor modeling can be considered a huge success.

The three main types of factor models are macroeconomic factor models, fundamental factor models and statistical factor models [5]. In a macroeconomic factor model, the factors are defined via economic theory and they are observed in addition to, and externally to, the security returns data. In a fundamental factor model, the aim is to find observable asset characteristics, e.g. financial ratios, capable of explaining the behavior of market stock prices, that are often extrinsic to the asset time-series.

The explanatory fundamental and economic variables can be highly correlated with each other, which may cause multicollinearity of factors. Returns predicted by a fundamental factor model may then be more correlated than the observed returns, which is the main reason for the inclusion of specific risk components in a factor model.

Classical factor models include only a handful of factors. One of the best-known factor model in the literature is the Capital Asset Pricing Model (CAPM), which assumes that a single risk factor, the market, drives returns in a portfolio of assets [6]. Recent increase in computational power has enabled development of models with a large set of factors. A number of factor models have extended this view [7, 8]. Today, factor models are popular in market risk modeling, e.g., the Barra models [9] depend on hand-picked market factors to explain behavior of the analyzed financial instruments.

Statistical factor models are a commonly-used alternative for fundamental and macroeconomic factor models. In a statistical factor model, factors are extracted from asset returns, and the set of factors may be large. The principal component analysis (PCA) is an example of a statistical technique for finding factors from asset time-series [10].

PCA works well when the analyzed time-series are highly correlated, which may indicate the presence of a common driver. Applications of PCA include models of interest rate term structure, credit spreads, futures, and volatility forwards. It is often the case that several principal components have an intuitive financial interpretation. In ordered highly-correlated systems, the first principal component captures an almost parallel shift in all variables and is generally labeled the common trend component. The second principal component captures an almost linear tilt in the variables, while the higher order principal components capture changes that are quadratic, cubic and so forth [11]. In the equity markets, the higher order principal components may often, but certainly not always, be interpreted as market movements caused by different investment style tilts.

Principal components found using PCA provide consistent estimators of the true latent factors in the limit of both time and cross-sectional size approaching infinity [12]. It can be shown that this extends consistent estimation of the classical factor model with non-correlated errors to approximate factor models with cross-correlated and sectionally-correlated error terms [12].

### 1.3 Choice of factors

The choice of factors clearly influences the ability of a factor model to explain investment risk of a portfolio, in particular when the factor model consists of only a few carefully-chosen factors. If the number of factors is large compared to the number of time-series analyzed, it may not much matter which factors are chosen as long as the factors span a sufficiently large sample space. Even then, relative importance of factors is of interest in risk management.

There are situations in which the use of a larger number of time-series may actually result a worse factor estimate than a smaller number of time-series [13]. A significant amount of recent literature has been devoted to addressing the issue of consistent estimation under conditions where the number of time-series is large compared to the length of time-series [14, 15].

Now, imagine that one is given a set of factors that are in no way fitted to the problem in question. Would this, rather arbitrary set of factors enable description, or better yet, reduction of the data to a smaller set of factors? It is not clear how well an arbitrary set of factors would enable analysis—or at least description—of the risk. This is the issue that we analyze in this study: take a random set of factors and see whether it enables reproduction of the data and its interdependencies.

### 1.4 Correlation structure and random matrices

Analysis of risk in an investment portfolio requires that risks of individual instruments are combined into the risk of the portfolio. This can be accomplished using dependence structures, e.g., correlation matrix. It is no coincidence that dependence structures of financial time-series are central in modern investment theory [16].

The recent explosion of available data has brought new issues with time-series analysis [14]. To get around these issues, new methods of analysis are needed. One such tool is the random matrix theory [17], which can be used to alleviate the issues of “Big data” [14]. For example, there are issues of correlation matrix estimation when there are more time-series than data points in each time-series, and the random matrix theory can be used to improve empirical estimates of correlation matrices [14]. In the random matrix theory, things often get less complex when dimension of the problem increases.

Another important feature of random matrix theory is universality. Most of the eigenvalue spectrum of correlation matrix for the Standard & Poor’s 500 Index (S&P 500) equities is part of the continuous eigenvalue bulk, only about 20 eigenvalues of 500 total are not [18]. The bulk of the power spectrum of the S&P 500 returns is very similar to that produced by Gaussian Orthogonal Ensemble [18], in which the asymptotic eigenvalue distribution is given by the Marchenko-Pastur law [19]. For this reason, it has been argued that eigenvalues in the bulk do not carry information [18], however, this view has recently been challenged [20].

Most eigenvalues in the spectrum of the cross-correlation matrix of stock price changes agree surprisingly well with universal predictions of random matrix theory [21]. This enables construction of a model with a spectrum that exhibits the features found in S&P 500 spectrum: a few large eigenvalues and a bulk part [22]. The 20 eigenvalues not part of the bulk may correspond to the market movements and sectors [18], but most of the eigenvalue spectrum does not appear to correspond to linear factors. For a more detailed discussion of eigenvalues in factor models, see [20, 23].

There also is the chicken-and-egg problem associated with factors: factors exist because stocks are correlated; stocks are correlated because of a common factor impacting them [22]. The apparent presence of factors is a consequence of the collective, bottom-up effect of the underlying time-series [22].

A naive use of the sample covariance matrix has drawbacks [24]. Shrinkage of the sample covariance matrix toward a structured or a constant matrix can be used to reduce extremal estimation errors [24]. Marchenko-Pastur law can be used to improve empirical estimates of correlation matrices further [15].

Given the above examples, it is clear that the random matrix theory can be used for analyzing financial time-series. We make use of random matrices to develop a factor model and to analyze the properties of correlation matrix preservation in a generic linear factor model.

### 1.5 The random projection

A good starting point for our analysis, and for developing a random factor model is the random projection method [25, 26]. The random projection allows one to reduce dimensionality of the investigated problem, often substantially, while preserving the structure of the problem. The random projection consists of a projection of data to a lower-dimensional space by a random matrix. The random projection method has been used, e.g., to reduce the complexity of the data for classification purposes [27], for structure-preserving perturbation of confidential data in scientific applications [28], for data compression [25], for compression of images [29], and in the design of approximation algorithms [30].

The previous literature has compared the performance of the random projection method with PCA, and found situations in which the random projection method performs comparably or even better than PCA. In the compression of image data and in text clustering, random projection method performed significantly better than PCA [25]. Random projection compares favorably with PCA, although PCA is more accurate with small number of dimensions [31].

In text clustering, a PCA-based method provides better accuracy with small number of dimensions, while with high number of dimensions the random projection method dominated [32]. In the analysis of five image data sets and five micro array data sets, PCA dominated with a small number of dimensions but its performance deteriorated when the dimensions of the data increased, while random projection dominates at high number of dimensions (cross-over occurs at 15–150 dimensions depending on the data set) [33]. The above results differ from the factor model setting in important ways, and the results cannot directly be applied to factor modeling. Still, they suggest that the random projection-based methods may be usable instead of PCA also in factor modeling.

### 1.6 This study

It is largely an open question whether and how well randomly-chosen factors can be used in a linear factor model to describe large data sets. This is the issue we approach in this study. For this purpose, we develop a factor model based on randomly-chosen factor time-series, the random factor model. We show that randomness of factors has certain desirable properties, such as well-defined probabilistic limits on the accuracy of the factor representation. In addition, randomly-chosen factors are almost orthogonal with high probability, and with a proper normalization, they are expected to be orthonormal. We also show how the random factor model converges toward the modeled data when the number of factors increases, and that a random factor model preserves pair-wise correlations well with high probability.

The article is structured as follows. In Section 2, we develop the random factor model based on the random projection method and derive theoretical results describing the model (more details can be found in S1 Appendix). For example, we show that randomness of factors enables derivation of theoretical results on the accuracy of the model.

From the econometric point of view, a random factor modeling can be viewed as a projection of time-series to a collection of factors that are almost non-autocorrelated and non-cross-correlated, as will be seen in Section 2.

As an application of the random factor model, Section 3 provides an analysis of the correlation matrix of the Russell 3,000 equity index using the factor models described in Section 2. We analyze the ability of random factor models to reproduce equity log-return time-series and their correlations and covariances. The reproduction of data in a random factor model is compared with a reproduction obtained using principal component analysis both at the individual time-series level and at the dependence structure level.

In Section 4, we compare different random factor models and show that the results, or rather their accuracy, are quite universal. In Section 5, we discuss the results and their possible implications.

In S1 Appendix, we prove an extended version of the Johnson-Lindenstrauss theorem appropriate for the random factor model. It gives probabilistic bounds on the accuracy of the correlation and covariance preservation in a random factor model.

## 2 Random factor model

### 2.1 Notations

Let *d* observations of time-series Z:N→R be viewed as a vector in *d*-dimensional space $R^{d}$, where each observation of the time-series corresponds to one coordinate of the vector. The set of *N* such time-series can be packed into matrix $X\in R^{d\times N}$, in which observations are in columns. We assume that the time-series data has been preprocessed, so that each time-series is averaged to zero, that is, ∑_*m*_ *X*_*mb*_ = 0 for each *b* = 1,…, *N*. We employ sample statistics in this study. Definitions for mean *μ*, variance *σ*^2^ and covariance *C* are

$$\begin{array}{c} \mu_{x}=\frac{1}{d}\sum_{m=1}^{d}x_{m},\;C_{x,y}=\frac{1}{d-1}\sum_{m=1}^{d}\left(x_{m}-\mu_{x}\right)\left(y_{m}-\mu_{y}\right),\;\sigma_{x}^{2}=C_{x,x},\; \end{array}$$ (1)

where $x,y\in R^{d}$. The central parameters used in this study are summarized in Table 1.

**Table 1:** The central parameters used.

Parameter	Description
*d*	Number of data points in each time-series
*N*	Number of time-series, e.g., equities
*k*	Number of factors

### 2.2 Linear factor models

A linear factor model describes the target data set as a loading-weighted sum of factors (e.g., [34]). Let $F=\left[F_{1}\;F_{2}\;...\;F_{k}\right]\in R^{d\times k}$ contain *d* observations of the factors *j* = 1, 2,…, *k*, $F_{j}\in R^{d\times 1}$. Then time-series $X_{b}\in R^{d\times 1}$, where *X*_*b*_ is *b*:th column of matrix *X*, *b* = 1,…, *N*, can be represented as a sum of products of factor loadings $L_{bj}\in R$ and factors *F*_*j*_, that is,

$$\begin{array}{c} X_{b}=\sum_{j=1}^{k}L_{bj}F_{j}+\epsilon_{b}, \end{array}$$ (2)

where *ϵ*_*b*_ is an idiosyncratic risk component. Since we collect the observations into columns of *X* and *F*, the formula (2) is written in a matrix form as *X* = *FL*^*T*^ + *ϵ*.

Factors in *F* may or may not be directly observable in the market data. For observed factor time-series, it suffices to project the data to factors to get loadings. For unobserved factor time-series, method such as PCA or another optimization method, e.g., Maximum Likelihood Estimation [11], is required to find the factors and their loadings.

### 2.3 Random projection

Random factors are here chosen using the random projection method. The key idea of random projection is based on the Johnson-Lindenstrauss lemma [25, 35]: if points in a high-dimensional space are projected onto a randomly selected subspace of suitably high dimension, then the distances between the points are approximately preserved. A suitably high-dimensional subspace has dimension proportional to log(*N*)/*ε*, where *N* is the number of time-series and *ε* the desired accuracy [36].

Random projection $Q:R^{d\times N}\to R^{k\times N}$ of matrix $D\in R^{d\times N}$ is a mapping defined by *Q*(*D*) = *BD*, where k,d,N∈N. Here matrix *B* is realization *B*(*ω*) of a random variable-valued *k* × *d* matrix. From this point on, we will not differentiate between random variables and their realizations and tacitly assume that the distinction can be inferred from the context.

A large variety of probability distributions can be used to construct projection matrix *B* (more on this in Section 3.3). The most obvious choice is to assume that matrix *B* is taken from the matrix-variate normal distribution with independent entries, that is, from *N*_*k*×*d*_(0, 1_*k*_ × 1_*d*_) [37]. Then each element is *N*(0, 1)-distributed and independent of other elements.

### 2.4 Random factor model

#### 2.4.1 Definition and properties

We define the random factor model (RFM) for data set $X\in R^{d\times N}$ via a projection. Strictly speaking, the matrix *P* is not a projection matrix since it typically does not satisfy the equality *P*^2^ = *P*. However, since its range is a lower dimensional subspace, we use the term “projection” also to describe *P*, in analogy with the definition of the term “random projection” in Section 2.3. $P:R^{d\times N}\to R^{d\times N}$,

$$\begin{array}{c} PX=aB^{T}BX, \end{array}$$ (3)

where *B* ∈ *N*_*k*×*d*_(0, 1_*k*_ × 1_*d*_) is a *k* × *d*-dimensional random variable, elements of which are independent and normally distributed, and *a* > 0 is a normalization constant. Mapping (3) can be interpreted as a linear factor model by setting

$$L=\frac{a}{a^{\prime }}X^{T}B^{T},$$ (4)

$$F=a^{\prime }B^{T}\;,$$ (5)

where *a*′ > 0 is a constant related to factor normalization, as discussed in Section 2.4.3. Then $F\in R^{d\times k}$ behaves as a matrix of *k d*-dimensional factor time-series. It is worth stressing that matrix *F* consists of random time-series that in no way depend on the data. $L\in R^{N\times k}$ is a matrix of *k* factor loadings for the *N* time-series.

Projection *P* can be factored as

$$\begin{array}{c} PX=FL^{T}. \end{array}$$ (6)

Defining ϵ* = *X* − *PX* yields an approximate factorization

$$\begin{array}{c} X=FL^{T}+\epsilon^{*} \end{array}$$ (7)

for data matrix X. We will analyze Eq (7), and in particular error term ϵ*, further in the following. Eq (7) shows that data matrix *X* can be approximately decomposed into a product of two components.

As an aside, let us mention that we could equally well have considered a random projection in the equity direction instead of the above time-series direction. This can be accomplished using a matrix *Q* = *aR*^*T*^ *R*, where *a* > 0 and $R\in R^{k\times N}$ is a random matrix, and then considering *XQ* as the projected matrix. This naturally leads to a factor model interpretation with a loading matrix $\frac{a}{a^{\prime }}R^{T}$ and a factor matrix *a*′*XR*^*T*^. In analogy to the earlier terminology, this model could be called *random loading model*. The properties proven later for the random projection *P* then immediately carry over to the projection *Q*, one merely needs to replace “*d*” with “*N*” in all of the results.

However, from the point of view of the time series, the two projection methods *PX* and *XQ* could behave differently. For instance, if there are more pronounced correlations between different equities at a fixed time than between the same equity at two different times, then one would expect to need larger values of *k* in the projection *XQ* than in the projection *PX* to reach the same level of accuracy in the approximation. It is also possible to apply both random projections simultaneously and study *PXQ* instead of *PX* or *XQ*. This double-sided projection would still have properties very similar to the one-sided projections, as long as the random matrices *B* and *R* are chosen independently of each other. Since the three alternatives are on a technical level very similar, we focus only on the choice *PX* in the following.

As the next step, we need to find a suitable constant *a* so that standard deviation, covariance and the expected value of the data are preserved, if possible. Under these conditions, ϵ* should be close to zero. Different choices of *a* yield slightly different properties for the RFM, but it turns out that we cannot satisfy all these requirements at the same time if we base matrix *B* on the normal distribution.

Here we concentrate on preserving the covariance matrix *C*_*x*,*y*_ in the projection. Then normalization constant *a* > 0 must be such that expectation with respect to *N*_*k*×*d*_(0, 1_*k*_ × 1_*d*_) is preserved, that is,

$$\begin{array}{c} E\left[C_{Px,Py}\right]=C_{x,y} \end{array}$$ (8)

for any zero-mean vectors $x,y\in R^{d\times 1}$. It is worth stressing that the expectation in Eq (8) is taken over random factor models, not over time-series *x* and *y*. Theorem 1.1 in S1 Appendix shows that this is possible but only if we choose $a=1/\sqrt{k(k+d)}$. Let *a* have this value from this point on.

The expected covariance between time-series *x* and *y* is then preserved, regardless of the number of factors used. Since $E\left[\sigma_{Px}^{2}\right]=E\left[C_{Px,Px}\right]=C_{x,x}=\sigma_{x}^{2}$, our choice of *a* also preserves time-series variance. This result shows that an RFM is expected to fulfill the consistency requirement of variance, that is, it shows that

$$\begin{array}{c} \lim_{d\to \infty }E\left[\sigma_{Px,d}^{2}\right]=\lim_{d\to \infty }\sigma_{x,d}^{2}=\sigma_{x,\text{pop}}^{2}, \end{array}$$ (9)

where $\sigma_{x,\text{pop}}^{2}$ is the population variance and $\sigma_{x,d}^{2}$ is the sample variance in dimension *d*. An application of the Jensen’s inequality implies that $E\left[\sigma_{Px}\right]\leq \sigma_{x}$, that is, volatility is not over-estimated.

Representation (7) always preserves the average of a zero-mean vector $x\in R^{d}$, that is, $E[\mu_{Px}]=0$. In contrast, the *m*:th observation *x*_*m*_ of time-series *x* has an expectation $E\left[{\left(Px\right)}_{m}\right]=\sqrt{k/(k+d)}x_{m}$ and a variance $(x_{m}^{2}+\left(d-1\right)\sigma_{x}^{2})/\left(d+k\right)$. For a small number of factors, mapping to (*Px*)_*m*_ will on average underestimate the original value *x*_*m*_ since $\sqrt{k/(k+d)}<1$. In the RFM, the number of factors *k* is not limited either by *N* or by *d*. In the limit of large number of factors, (*Px*)_*m*_ approaches *x*_*m*_, since

$$\begin{array}{c} \lim_{k\to \infty }E\left[{\left(Px\right)}_{m}\right]=\lim_{k\to \infty }\sqrt{k/(k+d)}x_{m}=x_{m}\;, \end{array}$$ (10)

and the standard deviation of (*Px*)_*m*_ is $O(\sqrt{d/(d+k)})$ and thus goes to zero when *k* → ∞. Hence, a RFM reproduces any vector $x\in R^{n}$ component-by-component in the limit of large number of number of factors, for *k* ≫ *d*. Thus *ϵ* of Eq (7) approaches zero when the number of factors increases.

The RFM is expected to reproduce mean, variance and covariance of time-series *x*. Component-wisely, the random factor model is expected to converge to the observed component values in the limit of large number of factors.

#### 2.4.2 Covariance preservation

Eq (8) does not state that each RFM always preserves the covariance matrix. Nevertheless, it is reasonable to assume that an RFM approximately preserves the covariance matrix. Next we will analyze how well an RFM will typically preserve the covariance matrix.

But first, it is worth recalling that Johnson-Lindenstrauss theorem [35, 36, 38] gives probabilistic bounds for the accuracy of distance preservation in the random projection. A number of versions of Johnson-Lindestrauss theorem have been proven, however, in all versions known to us, it is assumed that random variables have zero expectation.

Matrix $B^{T}B\in R^{d\times d}$ is a singular Wishart matrix (also known as an anti-Wishart matrix), which has non-zero expectation, *d* − *k* zero eigenvalues and *k* non-zero eigenvalues. Since matrix *B*^*T*^ *B* has non-zero expectation, it was not *a priori* clear if a Johnson-Lindenstrauss type theorem holds. Theorem 1.1 proven in S1 Appendix fills this gap for the present type of anti-Wishart matrices, and it also contains a detailed derivation of the above expectation values for an arbitrary value of the scaling parameter *a*.

We have collected in Corollary 1.2 in S1 Appendix the corresponding results for the choice which preserves the sample covariance matrices in expectation, for $a=1/\sqrt{k(k+d)}$. The precise control of fluctuations in the covariance estimates requires nontrivial combinatorial computations, given in S1 Appendix. As proven in the Corollary, for every *b* > 0 and non-random vectors $u,v\in R^{d}$, with *μ*_*u*_ = 0 = *μ*_*v*_, we have

$$\begin{array}{c} P\;[|C_{Pu,Pv}-C_{u,v}|\geq b]\leq \frac{8}{kb^{2}}\sigma_{u}^{2}\sigma_{v}^{2}\;. \end{array}$$ (11)

Inequality (11) gives bounds on the accuracy of covariance preservation for an arbitrary random factor model. Here probability is taken with respect to an ensemble of random factor models. Since our proof is based on the Chebyshev inequality, there could still be room for improvement in the estimate. Also, as noted in Remark 1.3 in S1 Appendix after the proof, the prefactor 8 in Eq (11) is not always optimal, and it could be reduced to 2 in the regime *d* ≫ *k*.

Hence, if $\sigma_{u}^{2},\sigma_{v}^{2}\leq 1$, the probability that covariance of vectors *u* and *v* is preserved in a random factor model more accurately than bound *b* is at least 1 − 8/(*kb*^2^), where *k* is the number of factors. In general, we can set *b* = *εσ*_*u*_*σ*_*v*_, with *ε* > 0, and also conclude that the accuracy, relative to the sample variance scale *σ*_*u*_ *σ*_*v*_, is at least *ε* with a probability of at least 1 − 8/(*kε*^2^). For the bound to be informative, it is necessary that *ε* > *k*^−1/2^.

The error in the covariance estimate decreases at least inversely with the number of factors in almost any random factor model. Given a sufficient number of factors, covariance of any two time-series can be approximated with an arbitrarily high accuracy using an RFM. This and the fact that random factors are in no way fitted to the data suggest that the typical accuracy of an RFM depends mainly on the number of factors *k*.

Corollary 1.2 in S1 Appendix also gives a bound on how accurately correlation between projected vectors is preserved. Correlation Corr(*u*, *v*) coincides with covariance *C*_*u*,*v*_ when *σ*_*u*_ = *σ*_*v*_ = 1. Then inequality (11) gives a lower bound on how well *C*_*Pu*,*Pv*_ approximates correlation between *u* and *v*.

These results can be summarized as a statement about the projected matrix *PX* as follows:

$$\begin{array}{c} P\;[\frac{1}{\sigma_{X_{b}}\sigma_{X_{c}}}\left|C_{{\left(PX\right)}_{b},{\left(PX\right)}_{c}}-C_{X_{b},X_{c}}\right|<\varepsilon ]\geq 1-\frac{8}{k\varepsilon^{2}}\;, \end{array}$$ (12)

valid for any *b*, *c* = 1, 2,…, *N* and *ε* > 0.

#### 2.4.3 Almost orthogonality

Orthogonality is a desirable property of a factor set. An orthogonalization procedure can be used to obtain an orthogonal factor set, but orthogonalization is computationally expensive. Fortunately, orthogonalization is not a necessary step in the RFM.

Given any two random factors (as defined above), their inner product is expected to be orthogonal, that is,

$$\begin{array}{c} E[\sum_{m=1}^{d}F_{mj}F_{mj^{\prime }}]={\left(a^{\prime }\right)}^{2}\sum_{m}E[B_{j^{\prime }m}B_{jm}]={\left(a^{\prime }\right)}^{2}d\delta_{j^{\prime },j}. \end{array}$$ (13)

This shows that with the choice $a^{\prime }=1/\sqrt{d}$, the factors *F*_*j*′_ and *F*_*j*_ are expected to be orthonormal as a consequence of the properties of normally distributed random variables. Hence, any two random factors are non-collinear with respect to each other.

Using Theorem 1.1 in S1 Appendix we can also compute the variance of the inner product,

$$\begin{array}{c} \text{Var}[\sum_{m=1}^{d}F_{mj}F_{mj^{\prime }}]=\frac{1}{d}\left(\delta_{j^{\prime },j}+1\right)\leq \frac{2}{d}. \end{array}$$ (14)

Higher cumulants approach zero even more rapidly, as can be seen by analyzing the cumulant generating function

$$\begin{array}{c} \ln E[e^{\lambda \sum_{m=1}^{d}F_{mj}F_{mj^{\prime }}}]=\{\begin{array}{c} -\frac{d}{2}\ln\left(1-2\frac{\lambda }{d}\right),\;\text{when}\;j=j^{\prime }\text{,}\\ -\frac{d}{2}\ln\left(1-\frac{\lambda^{2}}{d^{2}}\right),\;\text{otherwise,} \end{array} \end{array}$$ (15)

and its series expansion in λ. When *j* = *j*′, *n*th cumulant is of order *O*(*d*^1−*n*^). When *j* ≠ *j*′, cumulants are of order *O*(*d*^1−*n*^) for even *n* and zero otherwise. Convergence to the Normal distribution in the limit of large *d* then follows by the standard arguments (e.g., [39]).

Inner product matrix is approximately distributed as

$$\begin{array}{c} \sum_{m=1}^{d}F_{mj}F_{mj^{\prime }}∼\{\begin{array}{c} N\left(1,\sqrt{2/d}\right),\;\text{when}\;j^{\prime }=j,\\ N(0,\sqrt{1/d}),\;\text{otherwise}. \end{array} \end{array}$$ (16)

When *d* is large (≫1, 000), standard deviation is only a fraction of the expectation for diagonal elements. Fluctuations around zero are small for non-diagonal elements. The cumulant expansion shows that the factors are almost orthonormal even at a relatively low dimension.

In addition, the factors are on average orthogonal to the error term ϵ*: since ϵ* is an even polynomial of *B*:s and *F*_*j*_ is linear in them, we have $E[\sum_{m=1}^{d}F_{mj}\epsilon_{mb}^{*}]=0$ for all *j* = 1, 2,…, *k* and *b* = 1, 2,…, *N*.

### 2.5 Principal component analysis

PCA is a well-known technique which uses a linear transformation to form a simplified data set retaining the characteristics of the original data set [40]. In investment risk measurement, PCA is used to explain the covariance structure of a set of market variables through a few linear combinations of these variables. The general objectives of using PCA are to reduce the dimensions of covariance matrices and to find the main risk factors. The risk factors can then be used to analyze, e.g., the investment risk sources of a portfolio, or to predict how the value of the portfolio will develop.

Projection to principal components is most directly obtained using the singular value decomposition [41]. Given data matrix $X\in R^{d\times N}$, SVD decomposes it as $X=P_{L}DP_{R}^{T}$, where $P_{L}\in R^{d\times d}$ is matrix of left singular vectors, $P_{R}\in R^{N\times N}$ is matrix of right singular vectors, and $D\in R^{d\times N}$ is the rectangular diagonal matrix of singular values. PCA-based factor representation of *X* is given by *X* = *FL*^*T*^, where $L=P_{R}\in R^{N\times N}$ gives the factor loading matrix and $F=P_{L}D\in R^{d\times N}$ defines the factors of the *N* equities. When reducing the dimensions of the original dataset, the first *k* principal components with the largest eigenvalues are chosen to represent the original dataset. This yields an approximation of the data matrix using a subset of factors. A *k*-factor approximation of matrix *X* is given by *F*^(*k*)^(*L*^(*k*)^)^*T*^, where $L^{\left(k\right)}\in R^{N\times k}$ contains the first *k* factor loadings, $F^{\left(k\right)}\in R^{d\times k}$ contains the components of the first *k* factors from *P*_*L*_ *D*. It can be shown that in the mean-error sense, PCA gives the best linear *k*-factor approximation to matrix *X* (e.g., [42, 43]). Principal components correspond to directions along which there is most variation in the data. However, there are no guarantees that pair-wise distances are preserved in PCA.

PCA yields the relative importance of the most important risk sources (defined in factor matrix *F*) in an investment portfolio. The relative importance of risk factors is shown by the size of eigenvalues. The eigenvectors with highest eigenvalues correspond to the most important risk factors. Loadings then tell how much investment instruments depend on these factors.

Nevertheless, it should be stated that PCA aims to capture total variation, not correlations [40].

### 2.6 Comparison of factor models

Despite appearance, the RFM and PCA share many features. In both models, the data can be represented as *FL*^*T*^, where *L* contains *k* factor loadings and *F* defines *k* factor time-series with *d* observations. In PCA, the most important eigenvectors are found by choosing the largest eigenvalues. No such ordering is available for random vectors. A random vector is essentially as good as the next random vector as a factor.

The RFM has an *almost* orthonormal factors, while PCA yields strictly orthonormal factors. After finding the factors, both the RFM and PCA project the data to these vectors. The ways in which the RFM and PCA end up with representations of the data matrix are quite different: in PCA, data is projected along principal components (factors) and only the desired set of these projections (loadings) are kept. In the RFM, the data is projected along the random factors. The main difference is in the way that factors are chosen.

PCA requires *O*(*d*^2^*N*) + *O*(*d*^3^) operations, while the RFM requires *O*(*kdN*) operations, given the factor time-series. Since the number of factors is typically significantly smaller than the dimension of data, the RFM is computationally much more efficient than PCA.

We do not aim at proving the supremacy of the RFM over PCA. We rather use PCA as a yardstick against which the RFM is compared. It is worth remembering that there is no fitting to data in the RFM, so one could reasonably expect that PCA would surpass RFM in every respect in data experiments.

## 3 Application to the Russell 3,000 equity index

The Russell 3,000 index (ticker RAY in Bloomberg) measures the performance of the 3,000 largest US companies. The index represents about 98 percent of the investable US equity market. Here, we investigate how well a random factor model reproduces log-returns of the Russell 3,000 equities and their cross-correlations, and compare the results to those obtained using PCA.

For our analyses, we employ daily log-returns of Russell 3,000 equities from 2000-01-03 to 2013-02-20 (in total 3,305 observations). This interval contains several phases of the business cycle and certain special events, e.g., the crash of September 2008. Of the 3,000 constituent time-series in the index, we used a subset of 1,591 time-series with continuous daily data covering the entire period. To apply the analysis methods, the data is normalized by subtracting mean of each return time-series and by dividing by its standard deviation.

### 3.1 Reproduction of time-series

**Fig 1:**
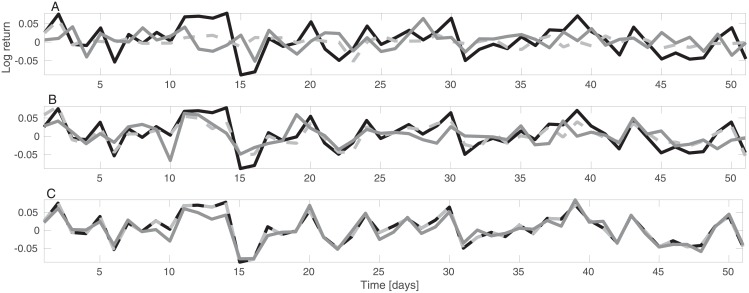
An example reproduction of logarithmic return time-series using the random factor model (dark grey curves) and PCA (dashed light grey curves) compared with the observed equity time-series (solid black curves) using 10 (top panel), 100 (middle panel), and 500 factors. The data is normalized to have zero average return, as describes in the text.

Fig 1 provides three examples of equity time-series reproduction using the RFM and PCA. The RFM (grey solid curve in Fig 1) provides a good reproduction of the single time-series even with a low number of factors. The accuracy of the reproduction improves with the number of factors: the agreement of the RFM and the data is very good with 500 factors.

As is demonstrated in Fig 1, random selection of factor time-series enables reproduction of (normalized) equity time-series, when the number of factors is sufficiently high. The number of factors is not limited to the number of time-series in the RFM, since the random factors do not necessarily span the entire space in which time-series may have values. Only in the limit of large number of factors is the entire space covered.

**Fig 2:**
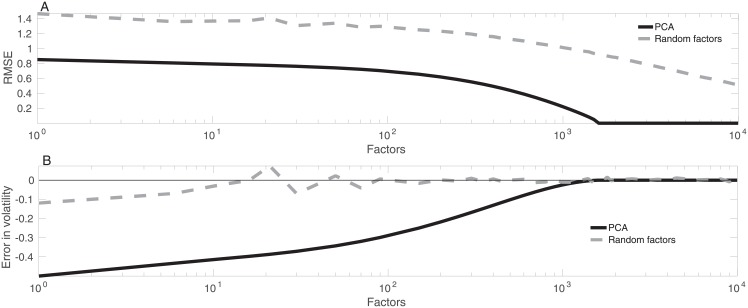
Accuracy of time-series representations. Error in time-series reproduction using the random factor model (dashed gray curve) and PCA (black solid curve) measured by RMSE (top panel), where curves are shown as functions of the number of factors. Error in reproduction of time-series volatility using the random factor model (dashed gray curve) and PCA (black solid curve) as a function of the number of factors (bottom panel). Errors are relative to the volatility of the time-series due to normalization.

Both PCA and the RFM provide good reproductions of the data (Fig 1), however, there are deviations from the data in each reproduction. In the root mean square error (RMSE) sense, PCA gives a better reproduction of the time-series than the RFM (Fig 2), as one would expect. RMSE in the reproduction of the entire data set is 0.79 in PCA vs 1.37 in the RFM with 10 factors (Fig 1).

### 3.2 Volatility

The RFM reproduces volatility of the time-series almost exactly even with a small number of factors, while in PCA volatility estimates improve pronouncedly with more factors (Fig 2). Since volatility of each time-series is normalized to 1 separately, accuracy of volatility reproduction is relative to volatilities of the underlying time-series in Fig 2.

In the RFM, error in volatility is 3.1 percent of volatility with ten factors. In PCA, error is 41.7 percent of volatility with ten factors. Modeling error is here defined as the difference *σ*_*model*_ − *σ*_*data*_ between volatility *σ*_*model*_ estimated from the modeled data and volatility *σ*_*data*_ computed from the original data. Accuracy increases until 1,000 factors is reached, after which essentially no error is observed in PCA. While the RFM reproduces the overall volatility of the equity time-series faithfully, it does not capture time-dependence of volatility particularly well (data not shown).

### 3.3 Correlation coefficient

**Fig 3:**
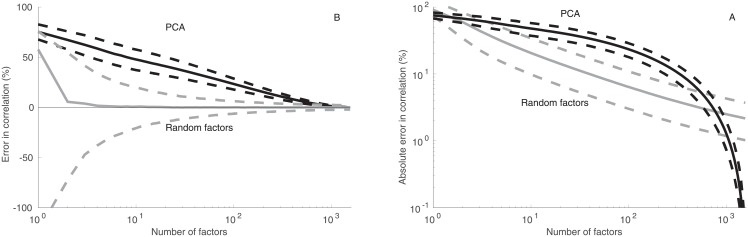
Accuracy of the correlation modeling. (A) Median error (solid gray curves; measured in percentage points) in correlation coefficient estimates in all pairs in the data-set estimated using 1,000 different random factor models, together with the 25th and 75th (dashed gray curves) percentiles of error in correlation estimates. The results are compared with the estimates of correlation based on PCA (solid black curve), together with the 25th and 75th (dashed black curves) percentiles. The results are shown as a function of the number of factors (abscissa). (B) Median absolute error of the random factor model (solid grey curve; measured in percentage points), together with the 25th and 75th percentiles (dashed grey curves); Median error in PCA (solid black curve) and the 25th and 75th percentiles (dashed black curves).

Fig 3 shows statistics on the accuracy of reproduction of correlation coefficients in all analyzed pairs of stocks. In the RFM, the median error converges rapidly to zero with only a few factors. The 25th and 75th percentiles of error converge toward zero when the number of factors increases. Together these three curves form a funnel (Fig 3) that rapidly converges toward zero. This shows that the typical accuracy of the correlation coefficient reproduction improves rapidly with the number of factors. Still, some noise persists even with the full set of 1,591 factors, for *k* = *d*.

In PCA, median error approaches zero only with around 1,000 factors, which is largely a consequence of PCA significantly underestimating volatilities of time-series. The 25th and 75th percentiles concentrate around the median away from zero in PCA.

Modeling error is here defined as the difference *c*_*model*_ − *c*_*data*_ between correlation *c*_*model*_ estimated from the modeled data and correlation *c*_*data*_ computed from the original data. Absolute modeling error is defined as the absolute difference |*c*_*model*_ − *c*_*data*_|.

Fig 3 shows results on absolute error in correlation coefficient as a function of the number of factors. In the RFM, correlation estimates converge toward the exact value when the number of factors is increased, however, convergence is less rapid than in analysis shown in Fig 3. This is a result of the fact that error can be in either direction in the RFM.

Compared with PCA, correlation estimates in the RFM converge significantly more rapidly toward the exact value. Since error is always in the same direction in PCA, there are no differences between absolute error and relative error in PCA-based analyses.

The RFM provides a more accurate description of correlation coefficients than PCA, when the number of factors is less than about 500. Noise inherent in the random factor model has the consequence that the error in correlation estimates does not disappear in the RFM even with the full set of 1,591 variables even though median estimate rapidly converges toward the observed correlation.

The cross-over to regime where PCA is more accurate occurs around 500–800 factors (Fig 3). When the number of factors is very high, PCA gives as good as or better estimates of correlation coefficients than the RFM. A factor model with such a large number of factors is of little use in practical applications.

### 3.4 Covariance

**Fig 4:**
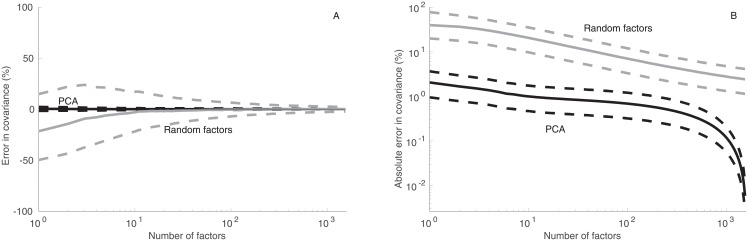
Accuracy of covariance estimation. Median error (left panel; solid gray curves; measure in percentage points) in covariance in all pairs in the data-set estimated using 1,000 different random factor models, together with 25th and 75th (dashed gray curves) percentiles of error in covariance estimates. The results are compared with the estimates of covariance based on PCA (solid black curve), together with the 25th and 75th (dashed black curves) percentiles. The results are shown as a function of the number of factors (abscissa). Median absolute error (right panel; solid gray curves; measure in percentage points) in covariance in all pairs in the data-set estimated using 1,000 different random factor models, together with the 25th and 75th (dashed gray curves) percentiles of error. The results are compared with the estimates of covariance based on PCA (solid black curve), together with the 25th and 75th (dashed black curves) percentiles.

The median error in covariance estimates converges rapidly toward zero in the RFM. The 25th and 75th percentiles form a funnel that converges toward zero when the number factors increases (Fig 4). Despite the fact that PCA is worse than the RFM in reproducing correlation coefficients, PCA gives a better reproduction of the covariance matrix (Fig 4).

### 3.5 Specific volatility and explanatory power

Above we saw that the RFM captured the average volatility of a time-series well. Still, PCA captured the specifics of data series more faithfully (measured by RMSE) than RFM, which suggests that specific volatility of a time-series is relatively large in the RFM. We can further elaborate on this by analyzing explanatory power. Define the explanatory power of a factor modeling by 1 − *σ*_*s*_/*σ*, where *σ* is the average volatility across all the securities in the data, and *σ*_*s*_ is the average asset-specific volatility not explained by the factor model [5].

**Fig 5:**
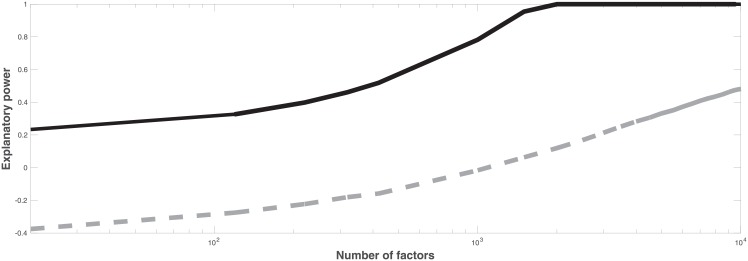
Explanatory power in factor modeling of data using PCA (black solid curve) and using the RFM (gray dashed curve).

Fig 5 compares explanatory power of PCA and an RFM and shows that PCA has a significantly higher explanatory power than the RFM. The result is in contrast to those presented in Fig 2. The difference is that Fig 5 shows (one minus) the ratio of the residual volatility and the total volatility, while Fig 2 shows that the RFM captures the time-averaged volatility well. Explanatory power of the RFM clearly increases with the number of factors, even when the number of factors increases toward the number of time-series. While an RFM reproduces the average volatility well, the specific volatility may be higher than the average volatility. This has the consequence that an RFM may even give negative explanatory power as a consequence of the definition of explanatory power.

### 3.6 Impact of the market factor

The risk in the equity market is often dominated by a single factor known as the market risk factor (e.g., Sharpe, 1964). To better analyze the other possible risk factors, we subtract the first principal component, corresponding to the market risk factor, from the data and reanalyze the remaining data (the “reduced data”).

**Fig 6:**
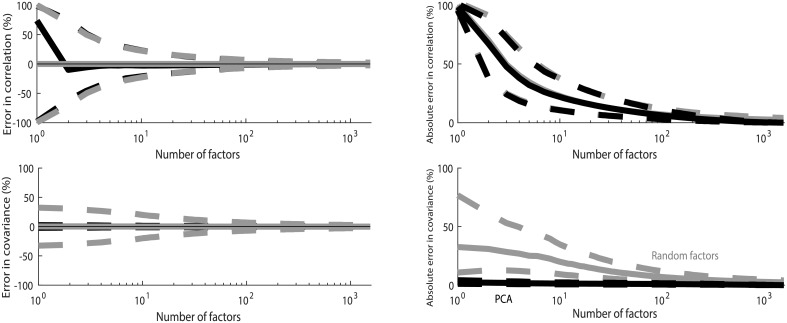
Analysis of the reduced data set in which the influence of the market is removed. Error and absolute error in correlation coefficient and in covariance estimates in an random factor model (gray curves) and in PCA (black curves). Solid lines are median estimates, dashed lines 25th and 75th percentiles.

Fig 6 shows that PCA becomes more accurate in reproducing the correlation coefficients when the impact of the market risk factor is removed from the data. Perhaps more surprisingly, reproduction of the data structure becomes equally accurate in the RFM and in PCA with respect to both error measures in the correlation coefficient (Fig 6). This suggests that the RFM and PCA contain equal amounts of information about the correlations.

**Fig 7:**
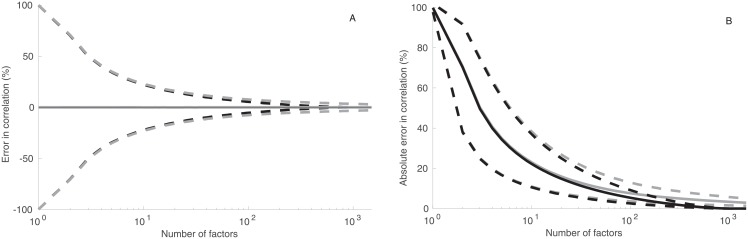
Reproduction of correlation coefficient in randomly-generated data. Error in correlation coefficient reproduction (left panel) of the random data using the random factor model (dashed gray curve) and PCA (black solid curve). Curves are shown as functions of the number of factors. Differences are in percentage points. The right panel shows absolute error in correlation reproduction of the random data using the random factor model (dashed gray curve) and PCA (black solid curve).

As a further check, we generated random data by sampling the normal probability distribution *N*(0, 1) repeatedly. Fig 7 shows that the accuracy of both the RFM and PCA is almost identical in this case. Comparison with Fig 5 shows that the accuracy of reproduction of the “reduced” Russell correlations does not significantly differ from the accuracy of the random data. This indicates that the fluctuations around the market risk factor are largely a product of independent “noise” contributions.

Removal of the market risk factor from the data also influences the accuracy of covariance reproduction. PCA is again more accurate than the RFM in covariance reproduction (Fig 6). In this case, the median error of covariance matrix reproduction does not deviate from zero in the RFM, and the 25th and 75th percentiles are almost symmetrically around x-axis.

## 4 Universality

A number of probability distributions have been found useful in the random projection method [2, 44]. Almost any probability distribution with zero mean, unit variance, and subgaussian tail fulfills the requirements of the Johnson-Lindenstrauss theorem [38]. These findings suggest that it may not matter much which probability distribution is used in the random projections. To find out whether this is the case in an RFM, we reanalyze the data using RFMs based on six different probability distributions. We have also discussed some lowest order effects of varying the probability distribution, as well as reasons why deviation from a Gaussian distribution leads only to small corrections, in Remark 1.4 after the proof in S1 Appendix.

### 4.1 Probability distributions

The six probability distributions that we employ here are two sparse matrix models of [44], a column-normalized Gaussian model, a row-normalized Gaussian model, the baseline Gaussian model (defined in Sec. 2.4) and a uniform model. In each case, the probability distribution is symmetric with respect to the origin and such that the expectation is zero. Each probability distribution has a subgaussian tail. These RFMs differ from the baseline Gaussian RFM only by the construction of the random projection matrix *B*, and by the normalization.

#### 4.1.1 Coin-flipping distributions

The simplest specification for random projection is the “random coin-flipping” algorithm of [44]. It is defined by choosing each element *B*_*pq*_ of matrix *B* randomly and independently according to rule: set *B*_*pq*_ = +1 with probability 0.5 and set *B*_*pq*_ = −1 with probability 0.5.

The second random projection that [44] proposes is based on a more sparse projection matrix defined by: set *B*_*pq*_ = + 1 with probability 1/6, set *B*_*pq*_ = 0 with probability 2/3 and set *B*_*pq*_ = −1 with probability 1/6. Again each element is chosen randomly and independently of the other elements. Based on these random projections, we can define two RFMs.

#### 4.1.2 Gaussian and uniform distributions

In addition to the baseline Gaussian RFM, we analyze two different RFMs based on the normal distribution. In the first RFM, matrix *B* is based on the spherical uniform distribution. The elements of matrix *B* are defined by

$$\begin{array}{c} B_{ml}=z_{ml}/Z, \end{array}$$ (17)

where *z*_*ml*_ ∼ *N*(0, 1) are independent and $Z=\sqrt{\sum_{p}{\left|z_{pl}\right|}^{2}}$. In this RFM, columns of matrix *B* are normalized in such a way that their length is exactly one.

Due to normalization of the columns of matrix *B*, diagonal elements of matrix $B^{T}B\in R^{d\times d}$ behave as in an orthogonal matrix. Then (*B*^*T*^ *B*)_*mm*_ = 1 for all *m* = 1, 2,…, *k*. Non-diagonal elements of *B*^*T*^ *B* have zero expectation and variance proportional to 1/*d* (Kaski, 1998). Hence, non-diagonal elements of *B*^*T*^ *B* are approximately distributed according to zero-mean normal distribution at a relatively low dimension. Therefore *B*^*T*^ *B* = 1 + *ϵ*, where $\epsilon \in R^{d\times d}$ has non-zero elements only on off-diagonal, E[ϵ]=0 and |E[ϵ]|<2/d. Matrix *B* is then almost orthonormal.

The second RFM based on the Gaussian probability distribution is a variation on the theme: Instead of column-normalization in the first model, rows of projection matrix *B* are normalized to unit length. This is the only difference between the two RFMs, but it is sufficient to require a different normalization constant.

The sixth considered RFM is defined by projection matrix B, which is based on the continuous uniform probability distribution. Each element in the projection matrix *B* is chosen randomly and independently from the uniform distribution on interval [−1, 1], that is, *B*_*mn*_ ∼ *U*(−1, 1) for each *m*, *n*.

### 4.2 Universality of distributions

**Fig 8:**
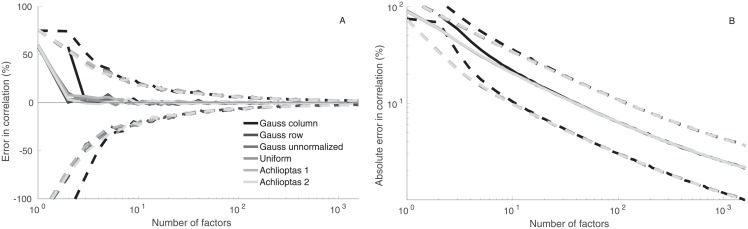
Comparison of six different projection matrix specifications. Solid lines are median estimates, dashed lines the 25th and 75th percentiles. Error in correlation coefficient estimates as functions of the number of factors in six models (left panel). Error is computed from the entire set of correlation pairs in 1,591 time-series. Absolute error in correlation coefficient estimates in the six models as functions of the number of factors (right panel).

Fig 8 shows that all six RFMs produce almost equally accurate results. To reduce noise, Fig 8 shows results averaged over 50 sample runs. When the number of factors exceeds 10, all RFMs produce almost identical median accuracy. The only deviation is the column-normalized Gaussian model, which deviates from the other RFMs when the number of factors is less than 5. All the other RFMs produce identical results also in this regime. The accuracy of the 25th and 75th percentiles mainly depends on the number of factors, not much on the way factors are generated or on the underlying probability distribution.

The results suggest that the details of how the projection matrix is specified are not that important. Almost any sufficiently regular construction of the random projection matrix (when properly normalized) produces a factor model, which preserves the approximate correlation structure. The main requirement here seems to be that matrix elements are chosen randomly and independently of other matrix elements. This supports the view that the RFM represents quite well how the bulk of factor models would reproduce the analyzed set of time-series.

## 5 Discussion

### 5.1 Random factors as explanatory factors

We set out to analyze the impact of random selection of factors on a linear factor model. Our interest was in whether and how randomness in the choice of factors impacts the reproduction of long equity time-series in a factor model, and the preservation of their interdependence.

We found that a random factor model (1) provides an almost orthonormal, non-multicollinear set of factors; (2) preserves the correlation matrix well; (3) preserves average volatility well; (4) has well-defined theoretical bounds on accuracy of the model; (5) preserves the structure of the data but not necessarily the details of single time-series. These are novel findings. One can confidently say that the random factor models performs significantly better than one would assume.

At the same time, it is worth pointing out the weak points of an RFM: (1) explanatory power of an RFM is quite low; (2) RMSE error of reproduction is higher than in PCA; (3) Only in the limit of infinite number of factors, an RFM is ensured to reproduce the original time-series perfectly; (4) an RFM does not provide factors capable of giving intuitive economic explanation of the data.

It may seem unlikely that a factor model with randomly-chosen factors could be used for any kind of factor modeling. One of the reasons for the ability of an RFM to capture the details of an equity time-series resides in the fact that random factors are, as a consequence of independence of elements, almost orthogonal to each other. The number of almost orthogonal vectors is higher in a higher-dimensional space, often attributed to [1]., which reduces the impact of the “curse of dimensionality” [45, 46] and thereby makes data representation more feasible. A suitably high number of random factors will then span a subspace sufficient to capture the return time-series at the desired accuracy.

If one were to try to model a set of time-series with a factor model with non-informative factors, the results would likely resemble those obtained with a random factor model. This obviously assumes that the set of factors would have low cross-correlations and little autocorrelation. Then our results would give an idea of the accuracy that one would reasonably expect to observe in the resulting model. In other words, our results give a lower bound on the accuracy of an informative factor model.

### 5.2 Universality

In a classical factor model, only a few factors are statistically significant. Then, explanatory power of each factor should be large. In a statistical factor model, a larger number of factors is often used, which has the consequence that a larger ambiguity in the choice of factors is encountered [24], as several different sets of factors may provide almost equally good fit to the data. In an RFM, each factor has only a small explanatory power, which suggests that a large number of factor sets provide essentially equally good descriptions of the data and its structure. This was observed in our computational experiments.

The number of random factors seems to be more important than fine-tuning of random factor time-series. The way an RFM is constructed is not important as long as the elements of projection *B* are independently drawn from a suitably regular probability distribution with zero expectation and subgaussian tails. We obtained almost identically accurate results regardless of the probability distribution used. These findings suggest that a kind of universality of RFMs is present, at least with respect to correlation coefficients. The results are largely dominated by a set of typical RFMs that have a rather similar accuracy of data reproduction. We have called this set of factor models the bulk.

The analysis of the proof of Theorem 1.1 in S1 Appendix (see Remark 1.4 in S1 Appendix) supports the view that universality is present with respect to probability distributions. The assumption that probability distribution is Gaussian is not necessarily required in the theorem. It suffices to assume independence of the random matrix elements, and it is likely that this requirement can be relaxed further.

## 6 Conclusions

A random factor model captures average volatility and reproduces correlations of the data with well-defined probabilistic error bounds, despite the fact that the factors are random and they are in no way fitted to the data. In the limit of large number of factors, the random factor model converges toward the exact reproduction of the data. Random factor modeling answers the question: how many factors does a generic linear factor model require to describe the correlation matrix of a dataset at a specific accuracy while preserving the average volatility of the time-series. Random factor models show universality of results: regardless of the method used to generate random factors, the results were essentially equally good.

## Supporting information

S1 Appendix Accuracy of the random factor approach. In S1 Appendix, we prove the main results used in the text for the mean and variance of the projection operators involved in the random factor models. We are mainly interested in controlling their asymptotic dependence for large values of the dimensions *k* and *d* of the factor matrix.
(TEX)
