# Correction: Random selection of factors preserves the correlation structure in a linear factor model to a high degree

**DOI:** 10.1371/journal.pone.0211610

**Published:** 2019-01-25

**Authors:** Antti J. Tanskanen, Jani Lukkarinen, Kari Vatanen

S1 Appendix was incorrectly included in LaTeX format. Please view the PDF version below.

## Supporting information

S1 Appendix Accuracy of the random factor approach. In S1 Appendix, we prove the main results used in the text for the mean and variance of the projection operators involved in the random factor models. We are mainly interested in controlling their asymptotic dependence for large values of the dimensions *k* and *d* of the factor matrix.[1]
(PDF)
