# Supplementary material for: Correction: Random selection of factors preserves the correlation structure in a linear factor model to a high degree
Source: PLoS One. 2019 Jan 25;14(1):e0211610. doi: 10.1371/journal.pone.0211610 (PMC6347217; doi:10.1371/journal.pone.0211610)
Supplement: S1 Appendix — In S1 Appendix, we prove the main results used in the text for the mean and variance of the projection operators involved in the random factor models. We are mainly interested in controlling their asymptotic dependence for large values of the dimensions k and d of the factor matrix.[1] (PDF) [file pone.0211610.s001.pdf]

S1 Appendix

Accuracy of the random factor approach

1 Provable bounds for the accuracy

In this Appendix, we prove the main results used in the text for the mean and variance of the projection operators involved in the random factor models. We are mainly interested in controlling their asymptotic dependence for large values of the dimensions k and d of the factor matrix. The exact dependence can be quite involved, see Eq (30), but in the Theorem below we provide a simpler bound which captures the correct asymptotics well for the values of interest here. The bound for the variance and Chebyshev's inequality straightforwardly lead to the bounds used in the text, as shown in Corollary 1.2 after the statement of the Theorem.

The derivation of the formula (30) for the variance of the sample covariance matrix involves some lengthy combinatorial estimates which here have been simplified by relying on suitable Wick polynomial representations of the matrix products. The first three items in the Theorem could equally well be proven by relying on the more standard Wick's product rule, i.e., Isserlis' theorem, which is valid for the Gaussian random variables defining the components of the random matrix B. The final item, however, becomes heavy to handle by this straightforward method and one has to be careful not to miss the cancellation of dominant terms in the expansion: The variance of a random variable X can be written using the moments of the variable as $\mathrm{Var}(X) = \mathbb{E}[X^2] - (\mathbb{E}[X])^2$ and hence one might expect it to be at least of the same order of magnitude as $\mathbb{E}[X]^2$. However, according to the results below, if $\mu_u = 0$, the term $\mathbb{E}[C_{Pu,Pv}]^2$ is proportional to $k^2(d+k)^2$ which is a factor k times larger than the bound for the variance given in the last item of the Theorem. This missing factor is also behind the $1/k$ decay factor in the last item of the Corollary.

The origin of the cancellation of leading terms becomes apparent once the expressions are written in terms of suitably chosen Wick polynomials of the random matrix elements. This representation has also the benefit of simplifying the computation of combinatorial factors leading to the expression (30). In addition, the Wick polynomial representation allows easier extension of the results to the case when the matrix elements do not have a Gaussian distribution, most notably since the cancellation of leading terms is related to the statistical independence of the components and not to Gaussianity of their distribution. However, we do not pursue the non-Gaussian case in detail here, but merely sketch some extensions of the results after the proof of the Theorem, in Remark 1.4.

Since Wick polynomials are not part of standard courses in probability, we have provided a summary of their definition and main properties in Sec 2 of the Appendix.

Theorem 1.1 *Suppose $k \geq 1$, $d \geq 2$ and that the matrix elements of the random matrix $B \in \mathbb{R}^{k\times d}$ are i.i.d. and $N(0,1)$-distributed. For some given $a > 0$ define a new random matrix $P \in \mathbb{R}^{d\times d}$ by $P := aB^TB$.*

Then for every non-random vectors $u, v \in \mathbb{R}^d$ all of the following results hold:

1. $\mathbb{E}[(Pu)_m] = aku_m$ *for all* m, *and* $\mathbb{E}[\mu_{Pu}] = ak\mu_u$.
2. $\mathrm{Var}((Pu)_m) = a^2k\left(u_m^2 + |u|^2\right)$ *for all* m.
3. $\mathbb{E}[C_{Pu,Pv}] = a^2k(d+k)C_{u,v} + a^2kd\mu_u\mu_v$.
4. *If* $\mu_u = \mu_v = 0$ *and* $d \geq 4$, *then* $\mathrm{Var}(C_{Pu,Pv}) \leq a^4 8k(k+d)^2\sigma_u^2\sigma_v^2$.

Corollary 1.2 *Suppose $k \geq 1$, $d \geq 4$ and that the matrix elements of the random matrix $B \in \mathbb{R}^{k\times d}$ are i.i.d. and $N(0,1)$-distributed. Set $a = [k(k+d)]^{-1/2}$ and define $P = aB^T B$.*

Then for every $b > 0$ and non-random vectors $u, v \in \mathbb{R}^d$, with $\mu_u = \mu_v = 0$, all of the following results hold:

1. $\mathbb{E}[(Pu)_m] = [k/(k+d)]^{1/2}\, u_m$ *for all m and* $\mathbb{E}[\mu_{Pu}] = 0$.
2. $\mathrm{Var}((Pu)_m) = \left(u_m^2 + (d-1)\sigma_u^2\right)/(d+k)$ *for all m.*
3. $\mathbb{E}[C_{Pu,Pv}] = C_{u,v}$.
4. $\mathbb{P}[|C_{Pu,Pv} - C_{u,v}| \geq b] \leq \frac{8}{kb^2}\sigma_u^2\sigma_v^2$.

Proof of the Corollary: With the added assumptions $\mu_u = \mu_v = 0$, the definition of sample variance yields the identities $|u|^2 = (d-1)\sigma_u^2$ and $|v|^2 = (d-1)\sigma_v^2$. Thus items 1, 2 and 3 are immediate corollaries using $a^2 = 1/(k(d+k))$. On the other hand, if we denote the standard deviation of $C_{Pu,Pv}$ by S, then by Chebyshev's inequality we have $\mathbb{P}[|C_{Pu,Pv} - C_{u,v}| \geq cS] \leq c^{-2}$ for any $c > 0$. Applying this with $c := b/S$ thus implies $\mathbb{P}[|C_{Pu,Pv} - C_{u,v}| \geq b] \leq S^2 b^{-2}$. Hence, item 4 of the Theorem implies the bound in the last item of the Corollary.

Proof of the Theorem: Assume k, d, B, a, P, u, v be given as in the theorem, and define $Z := Pu$, $Z' := Pv$ and $C := C_{Pu,Pv}$. Z_m, Z'_m, $m = 1, 2, \ldots, d$, and C are then all real-valued random variables. For any $m = 1, 2, \ldots, d$, the definitions yield

$$Z_m = (Pu)_m = a \sum_{n=1}^{d} \sum_{j=1}^{k} B_{jm} B_{jn} u_n \,. \tag{1}$$

Since B_{jm} are i.i.d. centered, normalized Gaussian, this implies

$$\mathbb{E}[Z_m] = a \sum_{n=1}^{d} \sum_{j=1}^{k} \mathbb{1}_{\{n=m\}} u_n = aku_m \,, \tag{2}$$

where $\mathbb{1}_{\{n=m\}}$ stands for an indicator function having value 1, when $n = m$, and 0 otherwise. Thus $\mathbb{E}[\mu_{Pu}] = \frac{1}{d}\sum_{m=1}^{d} \mathbb{E}[Z_m] = ak\mu_u$.

As motivated in the beginning of this S1 Appendix, we obtain a better control of the associated combinatorics by using Wick polynomial expansions. Their main properties are discussed in Sec 2. In particular, we rely here on the Wick polynomial expansion of arbitrary centered expectations of monomials of random variables, given in (39): for any product of random variables $x_1, x_2, \ldots, x_n$, with the corresponding index set $I = \{1, 2, \ldots, n\}$, one has

$$\prod_{i=1}^{n} x_i - \mathbb{E}\Bigg[\prod_{i=1}^{n} x_i\Bigg] = \sum_{\emptyset \neq E \subset I} \mathbb{E}\Bigg[\prod_{i\in I\setminus E} x_i\Bigg] {:}\prod_{i\in E} x_i{:} \,. \tag{3}$$

Therefore, as $\mathbb{E}[B_{jm}] = 0$, centering the variable Z_m yields the following simple Wick polynomial expansion:

$$Z_m - \mathbb{E}[Z_m] = a \sum_{n=1}^{d} u_n \sum_{j=1}^{k} {:}B_{jm} B_{jn}{:} \,. \tag{4}$$

The usefulness of Wick polynomials lies in the property that their products satisfy the same moment-to-cumulants expansion as simple products, with the additional rule

that *any partition with a cluster of indices inside one of the Wick polynomials will be missing* from the expansion. For instance, as shown in Sec 2, for any random variables x_1, x_2, x_3, x_4 we have

$$\mathbb{E}[{:}x_1x_2{:}\,{:}x_3x_4{:}] = \kappa[x_1,x_2,x_3,x_4] + \kappa[x_1,x_3]\kappa[x_2,x_4] + \kappa[x_1,x_4]\kappa[x_2,x_3]\,, \tag{5}$$

where κ denotes a cumulant. Here, for instance, $\kappa[x_1, x_3] = \mathrm{Cov}(x_1, x_3)$. Applying this to the B-variables yields

$$\mathbb{E}[{:}B_{jm}B_{jn}{:}\,{:}B_{j'm'}B_{j'n'}{:}] = \mathbb{1}_{\{j'=j,m'=m\}}\mathbb{1}_{\{j'=j,n'=n\}} + \mathbb{1}_{\{j'=j,n'=m\}}\mathbb{1}_{\{j'=j,n=m'\}}\,, \tag{6}$$

since for Gaussian random variables the fourth cumulant is equal to zero. Therefore, by (4) and (6), we have

$$\begin{aligned}\mathrm{Cov}(Z_m, Z'_{m'}) &= \mathbb{E}[(Z_m - \mathbb{E}[Z_m])(Z'_{m'} - \mathbb{E}[Z'_{m'}])]\\ &= a^2 \sum_{n',n=1}^{d} u_n v_{n'} \sum_{j',j=1}^{k} \mathbb{E}[{:}B_{jm}B_{jn}{:}\,{:}B_{j'm'}B_{j'n'}{:}]\\ &= a^2\left(k\mathbb{1}_{\{m'=m\}}\sum_{n=1}^{d} u_n v_n + k u_{m'} v_m\right) = a^2 k\left(\mathbb{1}_{\{m'=m\}} u\cdot v + u_{m'}v_m\right)\,,\end{aligned} \tag{7}$$

and thus, in particular,

$$\mathrm{Var}(Z_m) = \mathbb{E}[(Z_m - \mathbb{E}[Z_m])^2] = a^2 k\left(|u|^2 + u_m^2\right)\,. \tag{8}$$

Therefore, we have now proven the first two items of the Theorem.

The combinatorics gets progressively heavier in the remaining two items. Let us begin with the scalar product

$$\begin{aligned}Pu\cdot Pv = Z\cdot Z' &= \sum_{m=1}^{d} Z_m Z'_m\\ &= \sum_{m=1}^{d} z_m z'_m + ak\sum_{m=1}^{d}(u_m z'_m + v_m z_m) + a^2k^2\sum_{m=1}^{d} u_m v_m\,,\end{aligned} \tag{9}$$

where $z_m = Z_m - \mathbb{E}[Z_m] = Z_m - aku_m$, $z'_m = Z'_m - \mathbb{E}[Z'_m] = Z'_m - akv_m$ denote the centered variables. Taking an expectation and using (7) for $m' = m$ thus yields

$$\mathbb{E}[Pu\cdot Pv] = \sum_{m=1}^{d} a^2k\,(u\cdot v + u_m v_m) + a^2k^2 u\cdot v = a^2k(d+1+k)u\cdot v\,. \tag{10}$$

The definition of C reads explicitly

$$C = C_{Pu,Pv} = \frac{1}{d-1}\sum_{m=1}^{d}(Pu)_m(Pv)_m - \frac{d}{d-1}\mu_{Pu}\mu_{Pv} = \frac{1}{d-1}Z\cdot Z' - \frac{d}{d-1}\mu_Z\mu_{Z'}\,. \tag{11}$$

To compute its expectation, we still need to evaluate

$$\begin{aligned}\mathbb{E}[\mu_Z\mu_{Z'}] &= \mathrm{Cov}[\mu_Z,\mu_{Z'}] + \mathbb{E}[\mu_Z]\mathbb{E}[\mu_{Z'}] = \frac{1}{d^2}\sum_{m',m=1}^{d}\mathrm{Cov}[Z_m, Z'_{m'}] + a^2k^2\mu_u\mu_v\\ &= \frac{a^2k}{d^2}\sum_{m',m=1}^{d}\left(\mathbb{1}_{\{m'=m\}}u\cdot v + u_{m'}v_m\right) + a^2k^2\mu_u\mu_v = \frac{a^2k}{d}u\cdot v + a^2k(k+1)\mu_u\mu_v\,.\end{aligned} \tag{12}$$

Therefore,

$$\mathbb{E}[C] = \frac{a^2k}{d-1}(d+k)u\cdot v - \frac{d}{d-1}a^2k(k+1)\mu_u\mu_v = a^2k(d+k)C_{u,v} + a^2kd\mu_u\mu_v\,, \quad (13)$$

where in the last step we have used the identity $u \cdot v = (d-1)C_{u,v} + d\mu_u\mu_v$.

For the final result, let us assume in addition that $\mu_u = \mu_v = 0$. To avoid iterated Wick polynomials, let us begin with $C = \frac{1}{d-1}Z\cdot Z' - \frac{d}{d-1}\mu_Z\mu_{Z'}$ and express the two terms separately in Wick form. Namely, now

$$\mu_Z\mu_{Z'} = \frac{a^2}{d^2}\sum_{n',n=1}^{d} v_{n'}u_n \sum_{m',m=1}^{d}\sum_{j',j=1}^{k} B_{jm}B_{jn}B_{j'm'}B_{j'n'}\,, \quad (14)$$

where the product of four B-factors can be expanded using Wick polynomial expansion (3). Since only expectations of products of even number of B:s can be non-zero, we obtain

$$\begin{aligned} &B_{jm}B_{jn}B_{j'm'}B_{j'n'} - \mathbb{E}[B_{jm}B_{jn}B_{j'm'}B_{j'n'}] \\ &\quad = {:}B_{jm}B_{jn}B_{j'm'}B_{j'n'}{:} + \mathbb{E}[B_{jm}B_{jn}]\,{:}B_{j'm'}B_{j'n'}{:} + \mathbb{E}[B_{jm}B_{j'm'}]\,{:}B_{jn}B_{j'n'}{:} \\ &\qquad + \mathbb{E}[B_{jm}B_{j'n'}]\,{:}B_{jn}B_{j'm'}{:} + \mathbb{E}[B_{jn}B_{j'm'}]\,{:}B_{jm}B_{j'n'}{:} \\ &\qquad + \mathbb{E}[B_{jn}B_{j'n'}]\,{:}B_{jm}B_{j'm'}{:} + \mathbb{E}[B_{j'm'}B_{j'n'}]\,{:}B_{jm}B_{jn}{:} \\ &\quad = {:}B_{jm}B_{jn}B_{j'm'}B_{j'n'}{:} + \mathbb{1}_{\{m=n\}}\,{:}B_{j'm'}B_{j'n'}{:} + \mathbb{1}_{\{m'=n'\}}\,{:}B_{jm}B_{jn}{:} \\ &\qquad + \mathbb{1}_{\{j'=j\}}\big[\mathbb{1}_{\{m=m'\}}\,{:}B_{jn}B_{jn'}{:} + \mathbb{1}_{\{m=n'\}}\,{:}B_{jn}B_{jm'}{:} \\ &\qquad\quad + \mathbb{1}_{\{m'=n\}}\,{:}B_{jm}B_{jn'}{:} + \mathbb{1}_{\{n=n'\}}\,{:}B_{jm}B_{jm'}{:}\big] \end{aligned} \quad (15)$$

Therefore,

$$\begin{aligned} \mu_Z\mu_{Z'} - \mathbb{E}[\mu_Z\mu_{Z'}] &= \frac{a^2}{d^2}\sum_{n',n=1}^{d} v_{n'}u_n \sum_{m',m=1}^{d}\sum_{j',j=1}^{k} {:}B_{jm}B_{jn}B_{j'm'}B_{j'n'}{:} \\ &+ \frac{a^2}{d}\sum_{n',n=1}^{d} v_{n'}u_n \sum_{j=1}^{k}{:}B_{jn}B_{jn'}{:} + \frac{a^2}{d}\frac{d-1}{d}C_{u,v}\sum_{m',m=1}^{d}\sum_{j=1}^{k}{:}B_{jm}B_{jm'}{:}\,, \end{aligned} \quad (16)$$

where we have applied the assumptions $\mu_u = 0 = \mu_v$.

Similarly, since

$$\frac{1}{d-1}Z\cdot Z' = \frac{a^2}{d-1}\sum_{n',n=1}^{d} v_{n'}u_n \sum_{m=1}^{d}\sum_{j',j=1}^{k} B_{jm}B_{jn}B_{j'm}B_{j'n'}\,, \quad (17)$$

and by (15)

$$\begin{aligned} &B_{jm}B_{jn}B_{j'm}B_{j'n'} - \mathbb{E}[B_{jm}B_{jn}B_{j'm}B_{j'n'}] \\ &\quad = {:}B_{jm}B_{jn}B_{j'm}B_{j'n'}{:} + \mathbb{1}_{\{m=n\}}\,{:}B_{j'm}B_{j'n'}{:} + \mathbb{1}_{\{m=n'\}}\,{:}B_{jm}B_{jn}{:} \\ &\qquad + \mathbb{1}_{\{j'=j\}}\big[{:}B_{jn}B_{jn'}{:} + \mathbb{1}_{\{m=n'\}}\,{:}B_{jn}B_{jm}{:} \\ &\qquad\quad + \mathbb{1}_{\{m=n\}}\,{:}B_{jm}B_{jn'}{:} + \mathbb{1}_{\{n=n'\}}\,{:}B_{jm}B_{jm}{:}\big]\,, \end{aligned} \quad (18)$$

we obtain a Wick polynomial expansion

$$\begin{aligned} \frac{1}{d-1}Z\cdot Z' - \mathbb{E}\left[\frac{1}{d-1}Z\cdot Z'\right] &= \frac{a^2}{d-1}\sum_{n',n=1}^{d} v_{n'}u_n \sum_{m=1}^{d}\sum_{j',j=1}^{k} {:}B_{jm}B_{jn}B_{j'm}B_{j'n'}{:} \\ &+ \frac{a^2}{d-1}(2k+d+2)\sum_{n',n=1}^{d} v_{n'}u_n \sum_{j=1}^{k}{:}B_{jn}B_{jn'}{:} + a^2C_{u,v}\sum_{m=1}^{d}\sum_{j=1}^{k}{:}B_{jm}B_{jm}{:}\,. \end{aligned} \quad (19)$$

Combining the above results together finally yields a Wick polynomial expansion for the centered C,

$$\begin{aligned} C - \mathbb{E}[C] = {} & \frac{a^2}{d-1} \sum_{n',n=1}^{d} v_{n'} u_n \sum_{m=1}^{d} \sum_{j',j=1}^{k} {:}B_{jm} B_{jn} B_{j'm} B_{j'n'}{:} \\ & - \frac{a^2}{d(d-1)} \sum_{n',n=1}^{d} v_{n'} u_n \sum_{m',m=1}^{d} \sum_{j',j=1}^{k} {:}B_{jm} B_{jn} B_{j'm'} B_{j'n'}{:} \\ & + \frac{a^2}{d-1}(2k+d+1) \sum_{n',n=1}^{d} v_{n'} u_n \sum_{j=1}^{k} {:}B_{jn} B_{jn'}{:} \\ & + a^2 C_{u,v} \sum_{m=1}^{d} \sum_{j=1}^{k} {:}B_{jm} B_{jm}{:} - \frac{a^2}{d} C_{u,v} \sum_{m',m=1}^{d} \sum_{j=1}^{k} {:}B_{jm} B_{jm'}{:}\ . \end{aligned} \tag{20}$$

We use this formula to compute $\mathrm{Var}(C) = \mathbb{E}[(C - \mathbb{E}[C])^2]$. In the expanded formula terms containing a product of different degree Wick polynomials yield zero since whatever three pairings is used for the six B-factors, one of these pairings connects two elements inside the degree four Wick polynomial. Hence, for instance, $\mathbb{E}[{:}B_{j_1 m_1} B_{j_1 n_1} B_{j_1' m_1'} B_{j_1' n_1'}{:}{:}B_{j_2 m_2} B_{j_2 m_2'}{:}] = 0$.

The products of second order terms turn out to yield the dominant contribution. After first taking out a factor $a^4 d^{-2}(d-1)^{-2}$, it reads explicitly

$$\begin{aligned} & \mathbb{E}\Bigg[\bigg(d(2k+d+1) \sum_{n',n=1}^{d} v_{n'} u_n \sum_{j=1}^{k} {:}B_{jn} B_{jn'}{:} + d u \cdot v \sum_{m=1}^{d} \sum_{j=1}^{k} {:}B_{jm} B_{jm}{:} \\ & \quad - u \cdot v \sum_{m',m=1}^{d} \sum_{j=1}^{k} {:}B_{jm} B_{jm'}{:}\bigg)^2\Bigg] \\ & = d^2 (2k+d+1)^2 \sum_{n_1',n_1,n_2',n_2=1}^{d} v_{n_1'} u_{n_1} v_{n_2'} u_{n_2} \sum_{j_1,j_2=1}^{k} \mathbb{E}[{:}B_{j_1 n_1} B_{j_1 n_1'}{:}{:}B_{j_2 n_2} B_{j_2 n_2'}{:}] \\ & \quad + d^2 (u \cdot v)^2 \sum_{m_1,m_2=1}^{d} \sum_{j_1,j_2=1}^{k} \mathbb{E}[{:}B_{j_1 m_1} B_{j_1 m_1}{:}{:}B_{j_2 m_2} B_{j_2 m_2}{:}] \\ & \quad + (u \cdot v)^2 \sum_{m_1',m_1,m_2',m_2=1}^{d} \sum_{j_1,j_2=1}^{k} \mathbb{E}[{:}B_{j_1 m_1} B_{j_1 m_1'}{:}{:}B_{j_2 m_2} B_{j_2 m_2'}{:}] \\ & \quad + 2d^2 (2k+d+1) u \cdot v \sum_{n',n=1}^{d} v_{n'} u_n \sum_{j,j_2=1}^{k} \sum_{m_2=1}^{d} \mathbb{E}[{:}B_{jn} B_{jn'}{:}{:}B_{j_2 m_2} B_{j_2 m_2}{:}] \\ & \quad - 2d(2k+d+1) u \cdot v \sum_{n',n=1}^{d} v_{n'} u_n \sum_{j,j_2=1}^{k} \sum_{m_2 . m_2'=1}^{d} \mathbb{E}[{:}B_{jn} B_{jn'}{:}{:}B_{j_2 m_2} B_{j_2 m_2'}{:}] \\ & \quad - 2d (u \cdot v)^2 \sum_{j,j_2=1}^{k} \sum_{m,m_2 . m_2'=1}^{d} \mathbb{E}[{:}B_{jm} B_{jm}{:}{:}B_{j_2 m_2} B_{j_2 m_2'}{:}], \end{aligned} \tag{21}$$

which simplifies to

$$\begin{aligned}
&d^2(2k+d+1)^2k(|u|^2|v|^2+(u\cdot v)^2)+2d^2(u\cdot v)^2dk+2(u\cdot v)^2kd^2\\
&\quad+4d^2k(2k+d+1)(u\cdot v)^2-0-4d^2k(u\cdot v)^2\\
&\quad=d^2(2k+d+1)^2k|u|^2|v|^2\\
&\qquad+[d^2(2k+d+1)^2k+2d^3k+2d^2k+4d^2k(2k+d)](u\cdot v)^2\,. \qquad (22)
\end{aligned}$$

In the remaining products, the allowed pairings are in one-to-one correspondence to permutations where each factor in the left product is paired with the factor in its "permuted" position in the right product. Thus for order four terms we obtain a sum over $4! = 24$ terms, namely,

$$\begin{aligned}
&\mathbb{E}[{:}B_{j_1m_1}B_{j_1n_1}B_{j_1'm_1'}B_{j_1'n_1'}{:}\,{:}B_{j_2m_2}B_{j_2n_2}B_{j_2'm_2'}B_{j_2'n_2'}{:}]\\
&\quad=\mathbb{1}_{\{j_1=j_2,j_1'=j_2'\}}\left[\mathbb{1}_{\{m_1=m_2,n_1=n_2\}}+\mathbb{1}_{\{m_1=n_2,n_1=m_2\}}\right]\\
&\qquad\times\left[\mathbb{1}_{\{m_1'=m_2',n_1'=n_2'\}}+\mathbb{1}_{\{m_1'=n_2',n_1'=m_2'\}}\right]\\
&\qquad+\mathbb{1}_{\{j_1=j_2=j_1'=j_2'\}}\mathbb{1}_{\{m_1=m_2,n_1=m_2'\}}\left[\mathbb{1}_{\{m_1'=n_2,n_1'=n_2'\}}+\mathbb{1}_{\{m_1'=n_2',n_1'=n_2\}}\right]\\
&\qquad+\mathbb{1}_{\{j_1=j_2=j_1'=j_2'\}}\mathbb{1}_{\{m_1=m_2,n_1=n_2'\}}\left[\mathbb{1}_{\{m_1'=n_2,n_1'=m_2'\}}+\mathbb{1}_{\{m_1'=m_2',n_1'=n_2\}}\right]\\
&\qquad+\mathbb{1}_{\{j_1=j_2=j_1'=j_2'\}}\mathbb{1}_{\{m_1=n_2,n_1=m_2'\}}\left[\mathbb{1}_{\{m_1'=m_2,n_1'=n_2'\}}+\mathbb{1}_{\{m_1'=n_2',n_1'=m_2\}}\right]\\
&\qquad+\mathbb{1}_{\{j_1=j_2=j_1'=j_2'\}}\mathbb{1}_{\{m_1=n_2,n_1=n_2'\}}\left[\mathbb{1}_{\{m_1'=m_2,n_1'=m_2'\}}+\mathbb{1}_{\{m_1'=m_2',n_1'=m_2\}}\right]\\
&\qquad+\mathbb{1}_{\{j_1=j_2',j_1'=j_2\}}\left[\mathbb{1}_{\{m_1=m_2',n_1=n_2'\}}+\mathbb{1}_{\{m_1=n_2',n_1=m_2'\}}\right]\\
&\qquad\times\left[\mathbb{1}_{\{m_1'=m_2,n_1'=n_2\}}+\mathbb{1}_{\{m_1'=n_2,n_1'=m_2\}}\right]\\
&\qquad+\mathbb{1}_{\{j_1=j_2=j_1'=j_2'\}}\mathbb{1}_{\{m_1=m_2',n_1=m_2\}}\left[\mathbb{1}_{\{m_1'=n_2,n_1'=n_2'\}}+\mathbb{1}_{\{m_1'=n_2',n_1'=n_2\}}\right]\\
&\qquad+\mathbb{1}_{\{j_1=j_2=j_1'=j_2'\}}\mathbb{1}_{\{m_1=m_2',n_1=n_2\}}\left[\mathbb{1}_{\{m_1'=n_2',n_1'=m_2\}}+\mathbb{1}_{\{m_1'=m_2,n_1'=n_2'\}}\right]\\
&\qquad+\mathbb{1}_{\{j_1=j_2=j_1'=j_2'\}}\mathbb{1}_{\{m_1=n_2',n_1=m_2\}}\left[\mathbb{1}_{\{m_1'=m_2',n_1'=n_2\}}+\mathbb{1}_{\{m_1'=n_2,n_1'=m_2'\}}\right]\\
&\qquad+\mathbb{1}_{\{j_1=j_2=j_1'=j_2'\}}\mathbb{1}_{\{m_1=n_2',n_1=n_2\}}\left[\mathbb{1}_{\{m_1'=m_2,n_1'=m_2'\}}+\mathbb{1}_{\{m_1'=m_2',n_1'=m_2\}}\right]. \qquad (23)
\end{aligned}$$

Therefore,

$$\begin{aligned}
&\sum_{j_1',j_1,j_2',j_2=1}^{k}\mathbb{E}\Big[{:}B_{j_1m_1}B_{j_1n_1}B_{j_1'm_1'}B_{j_1'n_1'}{:}\,{:}B_{j_2m_2}B_{j_2n_2}B_{j_2'm_2'}B_{j_2'n_2'}{:}\Big]\\
&\quad=k^2\Big\{\left[\mathbb{1}_{\{m_1=m_2,n_1=n_2\}}+\mathbb{1}_{\{m_1=n_2,n_1=m_2\}}\right]\times\left[\mathbb{1}_{\{m_1'=m_2',n_1'=n_2'\}}+\mathbb{1}_{\{m_1'=n_2',n_1'=m_2'\}}\right]\\
&\qquad+\left[\mathbb{1}_{\{m_1=m_2',n_1=n_2'\}}+\mathbb{1}_{\{m_1=n_2',n_1=m_2'\}}\right]\times\left[\mathbb{1}_{\{m_1'=m_2,n_1'=n_2\}}+\mathbb{1}_{\{m_1'=n_2,n_1'=m_2\}}\right]\Big\}\\
&\quad+k\Big\{\mathbb{1}_{\{m_1=m_2,n_1=m_2'\}}\left[\mathbb{1}_{\{m_1'=n_2,n_1'=n_2'\}}+\mathbb{1}_{\{m_1'=n_2',n_1'=n_2\}}\right]\\
&\qquad+\mathbb{1}_{\{m_1=m_2,n_1=n_2'\}}\left[\mathbb{1}_{\{m_1'=n_2,n_1'=m_2'\}}+\mathbb{1}_{\{m_1'=m_2',n_1'=n_2\}}\right]\\
&\qquad+\mathbb{1}_{\{m_1=n_2,n_1=m_2'\}}\left[\mathbb{1}_{\{m_1'=m_2,n_1'=n_2'\}}+\mathbb{1}_{\{m_1'=n_2',n_1'=m_2\}}\right]\\
&\qquad+\mathbb{1}_{\{m_1=n_2,n_1=n_2'\}}\left[\mathbb{1}_{\{m_1'=m_2,n_1'=m_2'\}}+\mathbb{1}_{\{m_1'=m_2',n_1'=m_2\}}\right]\\
&\qquad+\mathbb{1}_{\{m_1=m_2',n_1=m_2\}}\left[\mathbb{1}_{\{m_1'=n_2,n_1'=n_2'\}}+\mathbb{1}_{\{m_1'=n_2',n_1'=n_2\}}\right]\\
&\qquad+\mathbb{1}_{\{m_1=m_2',n_1=n_2\}}\left[\mathbb{1}_{\{m_1'=n_2',n_1'=m_2\}}+\mathbb{1}_{\{m_1'=m_2,n_1'=n_2'\}}\right]\\
&\qquad+\mathbb{1}_{\{m_1=n_2',n_1=m_2\}}\left[\mathbb{1}_{\{m_1'=m_2',n_1'=n_2\}}+\mathbb{1}_{\{m_1'=n_2,n_1'=m_2'\}}\right]\\
&\qquad+\mathbb{1}_{\{m_1=n_2',n_1=n_2\}}\left[\mathbb{1}_{\{m_1'=m_2,n_1'=m_2'\}}+\mathbb{1}_{\{m_1'=m_2',n_1'=m_2\}}\right]\Big\}\,. \qquad (24)
\end{aligned}$$

For the terms involving $Z'\cdot Z$ we also need restrictions of this result to cases where

$m_1 = m_1'$ or $m_2 = m_2'$:

$$\sum_{m_1=1}^{d} \sum_{j_1',j_1,j_2',j_2=1}^{k} \mathbb{E}\Big[{:}B_{j_1m_1}B_{j_1n_1}B_{j_1'm_1}B_{j_1'n_1'}{:}{:}B_{j_2m_2}B_{j_2n_2}B_{j_2'm_2'}B_{j_2'n_2'}{:}\Big]$$
$$= (k^2+k)\big[\mathbb{1}_{\{m_2'=m_2,n_2=n_1,n_2'=n_1'\}} + \mathbb{1}_{\{m_2'=n_1',m_2=n_2',n_2=n_1\}}$$
$$+ \mathbb{1}_{\{m_2'=n_2,m_2=n_1,n_2'=n_1'\}} + \mathbb{1}_{\{m_2'=n_1',m_2=n_1,n_2'=n_2\}}$$
$$+ \mathbb{1}_{\{m_2'=m_2,n_2=n_1',n_2'=n_1\}} + \mathbb{1}_{\{m_2'=n_2,m_2=n_1',n_2'=n_1\}}$$
$$+ \mathbb{1}_{\{m_2'=n_1,m_2=n_2',n_2=n_1'\}} + \mathbb{1}_{\{m_2'=n_1,m_2=n_1',n_2'=n_2\}}\big]$$
$$+ 2k\big[\mathbb{1}_{\{m_2'=n_1,m_2=n_2,n_2'=n_1'\}} + \mathbb{1}_{\{m_2'=n_1',m_2=n_2,n_2'=n_1\}}$$
$$+ \mathbb{1}_{\{m_2'=n_2',m_2=n_1,n_2=n_1'\}} + \mathbb{1}_{\{m_2'=n_2',m_2=n_1',n_2=n_1\}}\big]\,. \tag{25}$$

Then we can collect the three terms needed here which are

$$\sum_{m_1,m_2=1}^{d} \sum_{j_1',j_1,j_2',j_2=1}^{k} \mathbb{E}\Big[{:}B_{j_1m_1}B_{j_1n_1}B_{j_1'm_1}B_{j_1'n_1'}{:}{:}B_{j_2m_2}B_{j_2n_2}B_{j_2'm_2}B_{j_2'n_2'}{:}\Big]$$
$$= k(k+1)\big[d\mathbb{1}_{\{n_2=n_1,n_2'=n_1'\}} + \mathbb{1}_{\{n_1'=n_2',n_2=n_1\}} + \mathbb{1}_{\{n_2=n_1,n_2'=n_1'\}} + \mathbb{1}_{\{n_1'=n_1,n_2'=n_2\}}$$
$$+ d\mathbb{1}_{\{n_2=n_1',n_2'=n_1\}} + \mathbb{1}_{\{n_2=n_1',n_2'=n_1\}} + \mathbb{1}_{\{n_1=n_2',n_2=n_1'\}} + \mathbb{1}_{\{n_1=n_1',n_2'=n_2\}}\big]$$
$$+ 2k\big[\mathbb{1}_{\{n_1=n_2,n_2'=n_1'\}} + \mathbb{1}_{\{n_1'=n_2,n_2'=n_1\}} + \mathbb{1}_{\{n_2'=n_1,n_2=n_1'\}} + \mathbb{1}_{\{n_2'=n_1',n_2=n_1\}}\big]$$
$$= [(d+2)k(k+1)+4k]\mathbb{1}_{\{n_2=n_1,n_2'=n_1'\}} + 2k(k+1)\mathbb{1}_{\{n_2'=n_2,n_1'=n_1\}}$$
$$+ [(d+2)k(k+1)+4k]\mathbb{1}_{\{n_2=n_1',n_2'=n_1\}}\,, \tag{26}$$

$$\sum_{m_1,m_2,m_2'=1}^{d} \sum_{j_1',j_1,j_2',j_2=1}^{k} \mathbb{E}\Big[{:}B_{j_1m_1}B_{j_1n_1}B_{j_1'm_1}B_{j_1'n_1'}{:}{:}B_{j_2m_2}B_{j_2n_2}B_{j_2'm_2}B_{j_2'n_2'}{:}\Big]$$
$$= k(k+1)\big[d\mathbb{1}_{\{n_2=n_1,n_2'=n_1'\}} + \mathbb{1}_{\{n_2=n_1\}} + \mathbb{1}_{\{n_2'=n_1'\}} + \mathbb{1}_{\{n_2'=n_2\}}$$
$$+ d\mathbb{1}_{\{n_2=n_1',n_2'=n_1\}} + \mathbb{1}_{\{n_2'=n_1\}} + \mathbb{1}_{\{n_2=n_1'\}} + \mathbb{1}_{\{n_2'=n_2\}}\big]$$
$$+ 2k\big[\mathbb{1}_{\{n_2'=n_1'\}} + \mathbb{1}_{\{n_2'=n_1\}} + \mathbb{1}_{\{n_2=n_1'\}} + \mathbb{1}_{\{n_2=n_1\}}\big]\,, \tag{27}$$

and

$$\sum_{m_1,m_1',m_2,m_2'=1}^{d} \sum_{j_1',j_1,j_2',j_2=1}^{k} \mathbb{E}\Big[{:}B_{j_1m_1}B_{j_1n_1}B_{j_1'm_1'}B_{j_1'n_1'}{:}{:}B_{j_2m_2}B_{j_2n_2}B_{j_2'm_2'}B_{j_2'n_2'}{:}\Big]$$
$$= k^2\big[d^2\mathbb{1}_{\{n_1=n_2,n_1'=n_2'\}} + d\mathbb{1}_{\{n_1=n_2\}} + d\mathbb{1}_{\{n_1'=n_2'\}} + 1$$
$$+ d^2\mathbb{1}_{\{n_1=n_2',n_1'=n_2\}} + d\mathbb{1}_{\{n_1=n_2'\}} + d\mathbb{1}_{\{n_1'=n_2\}} + 1\big]$$
$$+ k\big[3d\mathbb{1}_{\{n_1'=n_2'\}} + 3d\mathbb{1}_{\{n_1'=n_2\}} + 3d\mathbb{1}_{\{n_1=n_2'\}} + 3d\mathbb{1}_{\{n_1=n_2\}} +$$
$$+ 2 + d^2\mathbb{1}_{\{n_1=n_2',n_1'=n_2\}} + d^2\mathbb{1}_{\{n_1'=n_2',n_1=n_2\}}\big]\,. \tag{28}$$

This yields

$$
\begin{aligned}
&\mathbb{E}\Big[\Big(d\sum_{n',n=1}^{d} v_{n'}u_n \sum_{m=1}^{d}\sum_{j',j=1}^{k} {:}B_{jm}B_{jn}B_{j'm}B_{j'n'}{:} \\
&\quad - \sum_{n',n=1}^{d} v_{n'}u_n \sum_{m',m=1}^{d}\sum_{j',j=1}^{k} {:}B_{jm}B_{jn}B_{j'm'}B_{j'n'}{:}\Big)^2\Big] \\
&= d^2k\left\{[(d+2)(k+1)+4]|u|^2|v|^2 + (u\cdot v)^2[(d+2)(k+1)+4+2(k+1)]\right\} \\
&\quad + d^2k(k+1)(|u|^2|v|^2+(u\cdot v)^2) - 2d^2k(k+1)(|u|^2|v|^2+(u\cdot v)^2) \\
&= d^2k[(dk+d+k+5)|u|^2|v|^2 + (dk+d+3k+7)(u\cdot v)^2]\,. \qquad (29)
\end{aligned}
$$

Adding the term to (22), and multiplying the result by $a^4d^{-2}(d-1)^{-2}$ yields

$$
\begin{aligned}
\mathrm{Var}(C) = \mathbb{E}[(C-\mathbb{E}[C])^2] &= \frac{a^4}{d^2(d-1)^2}d^2k\Big\{(2k+d+1)^2|u|^2|v|^2 \\
&\quad + (dk+d+k+5)|u|^2|v|^2 + [(2k+d+1)^2+2d+2+4(2k+d)](u\cdot v)^2 \\
&\quad + (dk+d+3k+7)(u\cdot v)^2\Big\} \\
&= \frac{a^4}{(d-1)^2}k\Big[(4k^2+d^2+5dk+5k+3d+6)|u|^2|v|^2 \\
&\quad + (4k^2+d^2+5dk+15k+9d+10)(u\cdot v)^2\Big] \\
&\le \frac{a^4}{(d-1)^2}2k|u|^2|v|^2(4k^2+d^2+5dk+10k+6d+8) \\
&= a^4 2k\sigma_u^2\sigma_v^2(4(k+d)^2-3d^2-3dk+10k+6d+8)\,. \qquad (30)
\end{aligned}
$$

If $d\ge 4$, we have $-3d^2-3dk+10k+6d+8\le 0$ for all $k\ge 1$. Hence, for $d\ge 4$, the above bound can be simplified to the form given in the Theorem, namely then

$$\mathrm{Var}(C) \le a^4 8k(k+d)^2\sigma_u^2\sigma_v^2\,. \qquad (31)$$

This concludes the proof of the theorem.

Remark 1.3 The exact bound in (30) can also be approximated in other ways. Choosing the normalization as in the Corollary, with $a^2 = 1/(k(d+k))$, and assuming $d \gg k$, we obtain an estimate $\mathrm{Var}(C) \lesssim 2\sigma_u^2\sigma_v^2/k$ with a reduction of the prefactor from 8 to 2. The bound stated in the Theorem becomes optimal in the opposite regime, when $k \gg d$.

Remark 1.4 The assumption about sufficiently fast decay of correlations, here taken to be i.i.d., between the matrix elements is important for the above phenomena to occur. However, the precise statistics of the distribution of each matrix element plays much less a role. For instance, consider instead of Gaussian $N(0,1)$-distributed matrix elements taking them from some other distribution which has finite moments up to order four. For example, suppose that the distribution of each $B = B_{jm}$ has mean zero, $\mathbb{E}[B]=0$, a variance $c_2 = \mathbb{E}[B^2]$ and a fourth cumulant $c_4 = \kappa[B,B,B,B]$. As shown below, the resulting changes to the first three items in the Theorem are then an introduction of an overall scale c_2 and relatively weak dependence on $b_4 := c_4/c_2^2$, the excess kurtosis of the distribution of B.

Explicitly, instead of Eq (6) we then have

$$\mathbb{E}[{:}B_{jm}B_{jn}{:}\,{:}B_{j'm'}B_{j'n'}{:}] \\ = c_2^2\big(\mathbb{1}_{\{j'=j,m'=m\}}\mathbb{1}_{\{j'=j,n'=n\}} + \mathbb{1}_{\{j'=j,n'=m\}}\mathbb{1}_{\{j'=j,n=m'\}} + b_4\mathbb{1}_{\{j'=j,m'=m=n=n'\}}\big)\,. \tag{32}$$

Therefore, we obtain

$$\mathbb{E}[(Pu)_m] = c_2 a k u_m\,, \tag{33}$$

$$\mathrm{Cov}((Pu)_m,(Pv)_{m'}) = c_2^2 a^2 k\left(\mathbb{1}_{\{m'=m\}} u\cdot v + u_{m'}v_m + b_4\mathbb{1}_{\{m'=m\}}u_m v_m\right)\,, \tag{34}$$

and, retracing the necessary steps of the above proof thus yields

$$\mathrm{Var}((Pu)_m) = c_2^2 a^2 k\left(|u|^2 + (1+b_4)u_m^2\right)\,, \tag{35}$$

$$c_2^{-2}\mathbb{E}[C_{Pu,Pv}] = a^2 k(d+k)C_{u,v} + a^2 k d\mu_u\mu_v + a^2 k b_4\left(1-\frac{1}{d}\right)\left(C_{u,v} + \frac{d}{d-1}\mu_u\mu_v\right)\,. \tag{36}$$

Therefore, the scaling preserving the mean covariance for centered time series with $\mu_u = 0 = \mu_v$ is then given by

$$a = \frac{1}{c_2\sqrt{k[d+k+b_4(1-1/d)]}}\,. \tag{37}$$

Thus the main effect of changing the distribution is a fairly obvious scaling which corresponds to normalization of the variance of B to one. The effect of the fourth cumulant is insignificant, unless it is at least as large as k and d. The third cumulant plays no role in the above computation; it will, however, affect the value of the variance of $C_{Pu,Pv}$. In fact, there are quite a few new terms introduced to the computation of $\mathrm{Var}(C_{Pu,Pv})$. All of these, however, are still expected to be subdominant to the Gaussian contribution, as long as the higher order cumulants are not comparable to k and d. As in the explicit example above, each higher order cumulant should merely introduce new restrictions reducing the combinatorial factors arising from the pairing partitions computed in the proof of the Theorem.

2 Wick polynomials

As motivated in the beginning of Sec 1, we obtain a better control of the associated combinatorics by using Wick polynomial expansions. Appendix A of [1] provides a quick summary of the definition and main properties of general Wick polynomials, and we refer to [2,3] for more detailed expositions of the general case and to [4] for the case with Gaussian random variables. For the benefit of the reader, we collect the most directly relevant discussion also here.

Wick polynomials are a regularization technique for powers of random variables, with an arbitrary joint distribution. Consider for instance n random variables x_i, $i = 1, 2, \ldots, n$, which have finite joint moments up to order n. Explicitly, if J is a sequence of the above labels and it has a length at most n, we denote the corresponding product as $x^J = \prod_{j\in J} x_j$ and assume that $\mathbb{E}[|x^J|] < \infty$. The assumption is clearly true for any n if (x_i) are jointly Gaussian.

Then for any sequence J as above we may "regularize" the monomial x^J by adding lower order powers to it with suitable weights which depend on the joint distribution, resulting in so called Wick polynomials, denoted by ${:}x^J{:}$. The Wick polynomial ${:}x^J{:}$ is a

polynomial of the random variables x_i, $i \in J$, and has x^J as its highest order term. The lower order terms are uniquely determined (see [3] for details) by requiring that

$$:x^J: = x^J - \sum_{E \subsetneq J} \mathbb{E}[x^{J \setminus E}] \, :x^E: \,, \tag{38}$$

where the sum goes over all proper subsequences E of J, including the empty sequence for which we set $:x^\emptyset: = x^\emptyset = 1$. If the joint distribution of (x_j) is Gaussian, Wick polynomials reduce to Hermite polynomials.

An immediate consequence of the defining property (38) is that a centered product of random variables may be expressed in terms of Wick polynomials using the formula

$$x^J - \mathbb{E}[x^J] = \sum_{\emptyset \neq E \subset J} \mathbb{E}[x^{J \setminus E}] \, :x^E: \,. \tag{39}$$

In particular for a collection of random variables which have a distribution close to a Gaussian or which are nearly independent, it is often useful to represent their moments using joint cumulants. This can be done via the moment-to-cumulants formula which states that, under the above assumptions, for any sequence J we have

$$\mathbb{E}[x^J] = \sum_{\pi \in \mathcal{P}(J)} \prod_{A \in \pi} \kappa[x_A] \,, \tag{40}$$

where the sum goes over the collection $\mathcal{P}(J)$ of partitions of the sequence J and $\kappa[x_A]$ denotes the cumulant of the random variables x_j, $j \in A$. For a partition $\pi \in \mathcal{P}(J)$, we call its elements, subsequences $A \in \pi$, *clusters* (a subsequence A and a partition π of a sequence J may respectively be identified with a subset and a partition of the enumerating label set $I = \{1, 2, \ldots, |J|\}$ of the sequence). In particular, each cluster is thus a non-empty subsequence of J. If the distribution of (x_i) is centered, then any partition containing a singlet $A = \{i\}$ has $\kappa[x_A] = \mathbb{E}[x_i] = 0$ and hence such partitions then do not contribute to the sum in (40). In addition, if the distribution of (x_i) is jointly Gaussian, we have $\kappa[x_A] = 0$ for any subsequence with $|A| > 2$. Therefore, for centered, jointly Gaussian distributions (40) reduces to the Wick's product rule (Isserlis' theorem)

$$\mathbb{E}[x^J] = \sum_{\pi \in \mathcal{P}_2(J)} \prod_{A \in \pi} \kappa[x_A] \,, \tag{41}$$

where the sum goes over the pairings $\mathcal{P}_2(J)$ of the sequence J, i.e., over partitions where each cluster has a size two. For any pairing $A = \{i, j\}$, $i \neq j$, the cumulant $\kappa[x_A]$ is in fact equal to the covariance $\mathrm{Cov}(x_i, x_j) = \mathbb{E}[x_i x_j]$.

The following result, directly following from Proposition 3.8 in [3], details the main regularizing property of Wick polynomials: Suppose $L \geq 1$ is given and consider a collection of $L + 1$ index sequences J', J_ℓ, $\ell = 1, \ldots, L$, such that their total length, $|J'| + \sum_\ell |J_\ell|$, does not exceed n. Consider the composite sequence I, formed by starting with J_1 and then appending each sequence J_ℓ, $\ell = 2, 3, \ldots, L$, and finally the sequence J'. Then the expectation of the corresponding product of Wick polynomials satisfies

$$\mathbb{E}\Big[\prod_{\ell=1}^{L} :x^{J_\ell}: x^{J'} \Big] = \sum_{\pi \in \mathcal{P}(I)} \prod_{A \in \pi} \left(\kappa[x_A] \mathbb{1}_{\{A \not\subset J_\ell \ \forall \ell\}} \right) \,. \tag{42}$$

The constraint determined by the characteristic functions on the right hand side of (42) amounts to removing from the standard moment-to-cumulant expansion (40) all partitions which have any clusters internal to one of the Wick polynomial index sets J_ℓ.

Partition	Clusters in J_1 or J_2?
$\{1,2,3,4\}$	
$\{1\},\{2,3,4\}$	J_1
$\{2\},\{1,3,4\}$	J_1
$\{3\},\{1,2,4\}$	J_2
$\{4\},\{1,2,3\}$	J_2
$\{1,2\},\{3,4\}$	J_1, J_2
$\{1,3\},\{2,4\}$	
$\{1,4\},\{2,3\}$	
$\{1\},\{2\},\{3,4\}$	J_1, J_2
$\{1\},\{3\},\{2,4\}$	J_1, J_2
$\{1\},\{4\},\{2,3\}$	J_1, J_2
$\{2\},\{3\},\{1,4\}$	J_1, J_2
$\{2\},\{4\},\{1,3\}$	J_1, J_2
$\{3\},\{4\},\{1,2\}$	J_1, J_2
$\{1\},\{2\},\{3\},\{4\}$	J_1, J_2

Table 1. (*left column*) **Partitions of the set** $\{1,2,3,4\}$**.** (*right column*) Those of the sets $J_1 = \{1,2\}$ and $J_2 = \{3,4\}$ which contain a cluster of the partition on the left.

As an explicit example, used also in the proof of Theorem 1.1, let us consider the case of four random variables x_i, $i = 1,2,3,4$, which need not be Gaussian. The general moment-to-cumulants formula gives $\mathbb{E}[x_1x_2x_3x_4]$ as a sum of products of cumulants corresponding to the partitions of the set of 4 elements, having altogether 15 terms none of which needs to be zero. In contrast, if we consider the expectation $\mathbb{E}[{:}x_1x_2{:}\,{:}x_3x_4{:}]$, which corresponds to $L = 2$, $J_1 = \{1,2\}$, $J_2 = \{3,4\}$, and $J' = \emptyset$ in (42), an explicit comparison given in Table 1 shows that only three of the 15 partitions can have a non-zero weight. Therefore, (42) implies that

$$\mathbb{E}[{:}x_1x_2{:}\,{:}x_3x_4{:}] = \kappa[x_1,x_2,x_3,x_4] + \kappa[x_1,x_3]\kappa[x_2,x_4] + \kappa[x_1,x_4]\kappa[x_2,x_3]\,. \tag{43}$$

It is also possible to verify this formula by a direct computation. At the lowest orders, moment-to-cumulants formula (40) yields the identities $\mathbb{E}[x_1] = \kappa[x_1]$ and $\mathbb{E}[x_1x_2] = \kappa[x_1,x_2] + \kappa[x_1]\kappa[x_2]$. Therefore, the lowest order Wick polynomials can be written using cumulants as

$${:}x_1{:} = x_1 - \kappa[x_1]\,, \quad {:}x_1x_2{:} = x_1x_2 - \kappa[x_1]x_2 - \kappa[x_2]x_1 + \kappa[x_1]\kappa[x_2] - \kappa[x_1,x_2]\,. \tag{44}$$

Substituting these results into $\mathbb{E}[{:}x_1x_2{:}\,{:}x_3x_4{:}]$, and then converting the remaining moments to cumulants using (40), results in a sum with over 50 terms involving products of cumulants. Most of these terms will cancel out each other, leaving the three terms on the right hand side of (43). The difference in the effort between these two methods for obtaining the identity (43) should now be apparent, and it served as one of the main motivations for our choice of writing the proof in terms of the less conventional Wick polynomial approach instead of direct application of Wick's rule stated in (41).

References

1. Lukkarinen J, Marcozzi M, Nota A. Summability of Connected Correlation Functions of Coupled Lattice Fields. Journal of Statistical Physics. 2018;171(2):189–206.

2. Peccati G, Taqqu M. Wiener Chaos: Moments, Cumulants and Diagrams: A survey with Computer Implementation. vol. 1. Springer Science & Business Media; 2011.

3. Lukkarinen J, Marcozzi M. Wick polynomials and time-evolution of cumulants. Journal of Mathematical Physics. 2016;57(8):083301.

4. Janson S. Gaussian Hilbert spaces. vol. 129. Cambridge university press; 1997.
